# Congress of neurological surgeons systematic review and evidence-based guidelines for the role of imaging in newly diagnosed WHO grade II diffuse glioma in adults: update

**DOI:** 10.1007/s11060-025-05043-8

**Published:** 2025-05-08

**Authors:** Chaitra Badve, Abraham Nirappel, Simon Lo, Daniel A. Orringer, Jeffrey J. Olson

**Affiliations:** 1 University Hospitals Cleveland Medical Center, Cleveland, USA; 2 Department of Radiology, Case Western Reserve University School of Medicine, Cleveland, OH USA; 3 Department of Radiation Oncology, University of Washington, Seattle, WA USA; 4 Department of Pathology, NYU Grossman School of Medicine, New York, NY USA; 5 Department of Neurosurgery, Emory University School of Medicine, Atlanta, GA USA

**Keywords:** Grade II diffuse glioma, Perfusion, Diffusion, Amino acid, Positron emission tomography

## Abstract

**Target population:**

Adult patients with suspected or histologically proven WHO Grade II diffuse glioma.

**Question 1:**

In adult patients with suspected or histologically proven WHO Grade II diffuse glioma, do advanced MRI techniques using magnetic resonance spectroscopy, perfusion weighted imaging or diffusion weighted imaging provide superior assessment of tumor grade, margins, progression, treatment-related effects, and prognosis compared to standard neuroimaging?

**Recommendation:**

Level II: The use of diffusion imaging and dynamic susceptibility contrast (DSC), dynamic contrast enhancement (DCE) and arterial spin labeling (ASL) sequences are suggested to differentiate WHO Grade II diffuse glioma from higher grade gliomas when this is not accomplished by T2 weighted and pre- and post-gadolinium contrast enhanced T1 weighted imaging.

Level III: The use of diffusion and perfusion is suggested for obtaining information in genomics, prognosis, and post treatment monitoring when this information would be of value to the clinician and is not obtained through other methods.

Level III: The use of MR Spectroscopy is suggested to differentiate WHO Grade II diffuse glioma from higher grade gliomas when this is not accomplished by standard MRI, perfusion and diffusion techniques and when such information would be of value to the clinician.

**Question 2:**

In adult patients with suspected or histologically proven WHO Grade II diffuse glioma, does molecular imaging using amino acid PET tracers provide superior assessment of tumor grade, margins, progression, treatment-related effects, and prognosis compared to standard neuroimaging?

**Recommendation:**

Level III: If not already evident by MRI studies, the addition of amino acid PET with FET and FDOPA as a tracer is suggested to help determine if a brain lesion is a low grade glioma or high grade glioma.

Level III: If the standard clinical prognostic parameters are unclear and novel PET tracers are available, the clinician may consider FET to assist in determination of prognosis in an individual with grade II diffuse glioma.

Level III: Clinicians may use FDOPA PET in addition to MRI if additional information is required for detection of tumor progression.

## Introduction

### Goals and rationale

In 2015, the CNS published a guideline on the use of imaging for diagnosis and assessment of for diffuse low grade gliomas [1]. These lesions generally represent infiltrative, slow growing, intra-axial, primary brain tumors. They typically occur in individuals in the second to fourth decade of life. The natural history of these tumors is usually slower growth over time than in their higher-grade counterparts, with some ultimately transforming to a higher grade tumor [2–5].

Early and accurate diagnosis of these tumors is important for subsequent appropriate management, and the contribution of imaging to both diagnosis and prognosis is important. These gliomas are typically identified on an anatomic MRI study as a non-enhancing mass lesion, hypointense on T1 weighted imaging and hyperintense on T2 and FLAIR imaging sequences. The previously published guidelines regarding imaging in low grade gliomas addressed these standard MRI techniques with an eye toward use of diffusion and perfusion weighted MRI, magnetic resonance spectroscopy and nuclear medicine based techniques such as positron emission tomography and single-photon emission computed tomography to provide additional information [1]. As per the recommendations of the National Academies of Medicine, formerly the Institute of Medicine, this manuscript is a response to the suggested periodic updates of a given clinical guideline [6].

As part of this update, it is recognized that, since the first publication of these guidelines in 2015, the terminology for classification of these tumors has been updated by the WHO in 2016 and again in 2021 [7, 8]. From review of the search parameters noted below, one will see that the majority of the time interval searched includes the period when the terminology was taken from the 2016 WHO update. Thus, the publications qualifying for use in this guideline update most often refer to WHO grade II diffuse glioma. More recently, and after completion of the search interval used for this guideline update, the WHO 2021 classification of brain tumors has been released. In it, these lesions are referred to as “WHO grade 2 adult-type diffuse glioma” and are comprised of “astrocytoma, IDH-mutant, WHO grade 2” and “oligodendroglioma, IDH-mutant and 1p/19q-codeleted, WHO grade 2” [8]. This newest terminology includes a narrower population of tumors than described in previous versions of these classification systems. Few publications from the search interval used in this guideline update use this 2021 WHO update terminology. Therefore, the publications discussed in the text of this guideline update use the terminology appropriate for the time interval for that manuscript, that being WHO grade II diffuse glioma. As this guideline update is being published after the release of the WHO 2021 update, the recommendations themselves include the updated terminology. As the newest WHO terminology addresses a narrower spectrum of tumors, the recommendations made in this current update remain applicable. Going forward the impact of the new WHO classification system on publications regarding diagnosis, therapy, and prognosis of this group of tumors will unfold and be reflected in future updates of this guideline.

### Objectives

The objective of this guideline is to update the ability of magnetic resonance and PET/radiotracer techniques to accurately diagnose and provide prognostic information about WHO grade II diffuse glioma.

## Summary of prior recommendations

The previous set of guidelines was written for adults with a newly diagnosed lesion suspected to be or histopathologically proven to be a low-grade glioma. Recommendations were made in answer to 3 questions and are paraphrased as follows: The first question asked: what is the optimal imaging technique to be used in the diagnosis of a suspected low-grade glioma? A level II recommendation was made stating that in patients with a suspected brain tumor, the minimum MRI exam should be an anatomic exam with both T2 weighted and pre- and post-gadolinium contrast enhanced T1 weighted imaging. A level II recommendation was also made stating that the addition of diffusion and perfusion weighted MR imaging could be used in the assessment of suspected low-grade gliomas, for the purposes of discriminating between tumor subtypes and detection of higher-grade diagnoses. Lastly, a level III recommendation was made to support the potential for magnetic resonance spectroscopy (MRS) and nuclear medicine methods including positron emission tomography and single-photon emission computed tomography imaging to offer additional diagnostic specificity [1].

The second question asked which imaging sequences or parameters best predict the biological behavior or prognosis for patients with low grade glioma? A level III recommendation stated that perfusion weighted imaging when obtained as a part of an imaging diagnostic evaluation can play a role in estimating prognosis [1].

A third question asked what is the optimal imaging technique to be used in the follow-up of a suspected, or biopsy proven, low grade glioma? A level II recommendation stated that in patients with a diagnosis of low-grade glioma, anatomic imaging sequences should include T2/ FLAIR MR sequences and T1 weighted imaging before and after the administration of gadolinium-based contrast. A level III recommendation noted that for astrocytic tumors, baseline and longitudinal elevations in tumor perfusion as assessed by dynamic susceptibility contrast perfusion MRI are associated with shorter time to tumor progression but can be difficult to standardize in clinical practice [1].

## Methodology

### Writing group and question establishment

The Joint Tumor Section of the AANS and CNS and CNS Guidelines Committee have prioritized an update of the evidence-based clinical guidelines for management of newly diagnosed WHO grade II diffuse gliomas. The writers represent a multi-disciplinary panel of clinical experts encompassing neurosurgery and neuroradiology. The methodology and findings of the previous guidelines were reviewed, and additional questions were developed to incorporate recent literature addressing the diagnosis and assessment of prognosis for this group of tumors using a PICO format. The methodology used to produce this guideline is the same as for the prior 2015 version using the *CNS Guideline Methodology* and guidance provided by PRISMA [1, 9].

### Literature search

The previous version of this guideline encompassed the dates from the beginning of January 1990 through the end of December 2012. In this update the PubMed and Embase databases were searched from January 1, 2013, through January 31, 2020. A combined search was performed for questions 1 and 2 pertaining to WHO grade II diffuse gliomas. The search strategies are as follow:

#### PubMed

(((Low grade glioma PET) OR (Low grade glioma MRI) OR (low grade glioma MR perfusion) OR (low grade glioma MR diffusion) OR (low grade glioma imaging prognosis) OR (Low grade glioma diagnostic test) OR (Low grade glioma diagnosis CT) OR (Low grade glioma diagnosis PET) OR (Low grade glioma diagnosis MRI)) AND (“2013/01/01”[PDAT]: “3000”[PDAT]))).

#### Embase

(‘nuclear magnetic resonance imaging’/de OR ‘magnetic resonance imaging’:ti,ab OR nmri:ti,ab OR mri:ti,ab OR mris:ti,ab OR ‘magnetic resonance tomography’:ti,ab OR ‘magnetization transfer imaging’:ti,ab OR ‘mr imaging’:ti,ab OR ‘nmr imaging’:ti,ab OR ‘magnetic resonance perfusion’/de OR ‘magnetic resonance perfusion’:ti,ab OR ‘mr perfusion’:ti,ab OR ‘diagnostic imaging’/de OR ‘diagnostic imaging’:ti,ab OR ‘imaging prognosis’:ti,ab OR ‘imaging prognoses’:ti,ab OR ‘diagnostic neuroimaging’:ti,ab OR ‘diffusion tensor imaging’/de OR ‘diffusion tensor imaging’:ti,ab OR ‘mr diffusion’:ti,ab OR ‘diffusion tensor magnetic resonance imaging’:ti,ab OR ‘diffusion tensor mri’:ti,ab OR ‘diffusion tensor tractography’:ti,ab OR ‘magnetic resonance diffusion tensor imaging’:ti,ab OR (‘low grade glioma’ AND (‘diagnostic test’ OR ‘diagnostic tests’)) OR ((‘computer assisted tomography’/de OR ‘cat scan’:ti,ab OR ‘computed tomographic scan’:ti,ab OR ‘computed tomography’:ti,ab OR ‘computed tomography scan’:ti,ab OR ‘computer tomography’:ti,ab OR ‘computerised axial tomography’:ti,ab OR ‘computerised tomography’:ti,ab OR ‘computerized axial tomography’:ti,ab OR ‘computerized tomography’:ti,ab) AND diagnos*) OR ((‘positron emission tomography’/de OR ‘positron emission tomography’:ti,ab OR ‘pet scan;’:ti,ab OR ‘positron emission tomographic scan’:ti,ab OR ‘positron tomography’:ti,ab OR ‘positron-emission tomography’:ti,ab) AND diagnos*)) AND (‘astrocytoma’/de OR ‘oligodendroglioma’/exp OR ‘low grade glioma’:ti,ab OR ‘low-grade glioma’:ti,ab OR ‘low grade gliomas’:ti,ab OR ‘low-grade gliomas’:ti,ab OR astrocytoma*:ti,ab OR oligodendroglioma*:ti,ab OR (‘glioma’/exp AND (‘low grade’:ti,ab OR ‘low-grade’:ti,ab))) NOT (‘animal’/exp NOT (‘animal’/exp AND ‘human’/exp)) NOT (‘editorial’/exp OR ‘letter’/exp OR ‘conference paper’/exp OR ‘case report’/de OR ‘conference abstract’/it OR ‘in vitro study’/exp) AND [english]/lim AND [1–1–2013]/sd NOT [3–2–2020]/sd AND (‘article’/it OR ‘article in press’/it OR ‘review’/it) NOT (‘juvenile’/exp NOT (‘juvenile’/exp AND ‘adult’/exp)).

## Inclusion/exclusion criteria

The 1661 citations derived from these searches were manually reviewed by the team with specific inclusion and exclusion criteria as outlined below. To be included in the guideline, a publication had to meet the following inclusion criteria:

- Peer-reviewed publications
- Clinical studies in adult patients with newly diagnosed LGG (LGG terminology was used according to the new WHO classification: WHO grade II diffused glioma)
- Clinical studies in adult patients with mixed new and recurrent/progressive LGG were included only if we could separate the outcomes for patients with newly diagnosed LGG from the other patients
- Each study reporting on at least five subjects
- Adult patients (> 18 years of age). Studies with mixed adult and pediatric populations are included if the adult cohorts could be isolated and analyzed separately
- Publications written in English

**Fig. 1:**
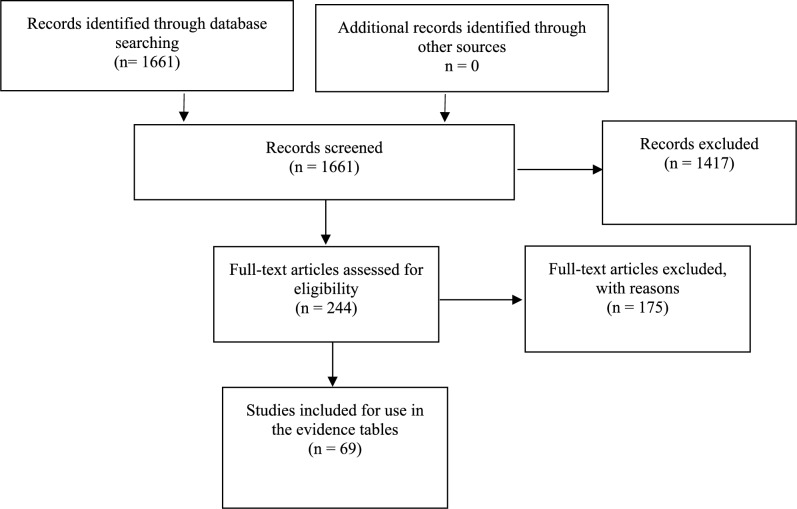
PRISMA diagram

Three independent reviewers considered the abstract data and accepted or set aside citations based on the information in the abstract. Full texts for those accepted citations were then obtained for careful review of information. If the manuscript still met inclusion criteria then it became eligible for inclusion in the evidence tables for these questions. Disagreements about inclusion or exclusion of citations or full text documents were resolved by consensus. When consensus could not be reached, the citation in question was not included. Citations that considered adult patients focusing on imaging in the diagnosis, and assessment of prognosis of LGG were considered. We allowed that manuscripts could focus on a comparison of imaging features of LGG with high grade glioma or other tumor types, as long as the data on the LGG could be analyzed separately by the reader. Abstracts that focused on a pediatric population, therapeutic studies, case reports noting imaging features of unusual tumor types, articles focusing on brainstem gliomas or spinal cord tumors, or those focusing on imaging and correlative histopathology markers as the primary subject were not included for review. This manual secondary review resulted in a list of 244 references that appeared best suited to answer the two questions, all 244 references were pulled for formal paper review and possible inclusion in evidence tables to help answer the questions in this section (see Fig. 1). The methodology used to produce this guideline is the same as for the prior 2015 version using the *CNS Guideline Methodology* and guidance provided by PRISMA [1, 9].

### Assessment for risk of bias

Following broad screening for relevance, two independent reviewers evaluated citations and full text screening of potentially relevant papers using a priori criteria for data extraction in a standardized manner. Our search generated a list of abstracts, which were screened, and those articles that addressed our identified questions underwent full independent review by the authors. Reviewers were critical in their assessment, specifically regarding trial design, such as randomization between imaging methods, blindedness, prospective character, size of study population, comparative baseline characteristics between study groups survivorship bias, selection bias, and appropriate statistical analyses of reported data. Disagreements were resolved by discussion of the points of contention between reviewers, using re-review of the manuscript until agreement was achieved. The methodology used to produce this guideline is the same as for the prior 2015 version using the *CNS Guideline Methodology* and guidance provided by PRISMA [1, 9].

### Rating quality of evidence

Both the quality of the evidence and the eventual strength of the recommendations generated by this evidence were graded according to a three-tiered system for assessing studies addressing diagnostic testing as approved by the American Association of Neurological Surgeons (AANS) and Congress of Neurological Surgeons (CNS) Joint Guidelines Review Committee on criteria (Table 1). Imaging studies that considered markers of diagnostic specificity were reviewed using these guidelines, considering a histopathological diagnosis as a ‘‘gold standard’’. In order to have class I evidence leading to level I recommendations regarding imaging, data must be from one or more well-designed clinical studies in a diverse population using a ‘‘gold standard’’ reference test. Well-designed clinical studies should include a blinded evaluation appropriate for the diagnostic applications and allow calculation of study intervention sensitivity, specificity, positive and negative predictive values, and where applicable, likelihood ratios. Class II evidence and level II recommendations require that evidence be provided by one or more well-designed clinical studies of a restricted population using a ‘‘gold standard’’ reference test in a blinded evaluation appropriate for the diagnostic applications and enabling the assessment of sensitivity, specificity, positive and negative predictive values, and, where applicable, likelihood ratios. For Class III evidence and/or a Level III recommendation, data is provided by expert opinion or studies that do not meet the criteria for the delineation of sensitivity, specificity, positive and negative predictive values, and, where applicable, likelihood ratios. The methodology used to produce this guideline is the same as for the prior 2015 version using the *CNS Guideline Methodology* and guidance provided by PRISMA [1, 9].

**Table 1:** Classification of evidence on diagnosis

Class of evidence	Definition
Class I Evidence	Evidence provided by one or more well-designed clinical studies of a diverse population using a “gold standard” reference test in a blinded evaluation appropriate for the diagnostic applications and enabling the assessment of sensitivity, specificity, positive and negative predictive values, and, where applicable, likelihood ratios
Class II Evidence	Evidence provided by one or more well-designed clinical studies of a restricted population using a “gold standard” reference test in a blinded evaluation appropriate for the diagnostic applications and enabling the assessment of sensitivity, specificity, positive and negative predictive values, and, where applicable, likelihood ratios
Class III Evidence	Evidence provided by expert opinion or studies that do not meet the criteria for the delineation of sensitivity, specificity, positive and negative predictive values, and, where applicable, likelihood ratios

From: https://www.cns.org/guidelines/guideline-development-methodology

Imaging studies that consider these same markers with respect to prognosis were reviewed considering five technical criteria (Table 2). If all five of these criteria are satisfied, the evidence is classified as Class I. If four out of five are satisfied, the evidence is Class II, and if less than 4 are satisfied, it is Class III:

- Was a well-defined representative sample of patients assembled at a common (usually early) point in the course of their disease?
- Was patient follow-up sufficiently long and complete?
- Were objective outcome criteria applied in a ‘‘blinded’’ fashion?
- If subgroups with different prognoses were identified, was there adjustment for important prognostic factors?
- If specific prognostic factors were identified, was there validation in an independent ‘‘test set’’ group of patients?

**Table 2:** Classification of evidence on prognosis

Class of Evidence	Definition
Class I Evidence	All 5 technical criteria above are satisfied
Class II Evidence	Four of five technical criteria are satisfied
Class III Evidence	Everything else

From: https://www.cns.org/guidelines/guideline-development-methodology

### Revision plans

In accordance with the Institute of Medicine’s standards for developing clinical practice guidelines and criteria specified by the National Guideline Clearinghouse, the task force will monitor related publications following the release of this document and will revise the entire document and/or specific sections. In addition, the task force will confirm within five years from the date of publication that the content reflects current clinical practice and the available technologies for the evaluation and treatment for patients with diffuse low grade glioma. The methodology used to produce this guideline is the same as for the prior 2015 version using the *CNS Guideline Methodology* and guidance provided by PRISMA [1, 9].

## Results

*Q1: In adult patients with suspected or histologically proven WHO Grade II diffuse glioma, do advanced MRI techniques using magnetic resonance spectroscopy, perfusion weighted imaging or diffusion weighted imaging provide superior assessment of tumor grade, margins, progression, treatment-related effects, and prognosis compared to standard neuroimaging?*

### Diffusion imaging

#### Study selection and characteristics

On full text-review, 16 articles concerning various diffusion techniques, all providing class III evidence were eligible for use in guideline creation as outlined in Table 3. Another 17 articles on multiparametric MR imaging studies including diffusion imaging as one of the techniques are included in separate table on multiparametric MR imaging (Table 6). Three articles concerning multi-modality imaging where MR diffusion imaging was investigated along with amino acid PET are included in the relevant PET table (Table 7). Thus, a total of 36 full text articles, all providing class III evidence concerning diffusion MR were included for guideline creation.

**Table 3:** Diffusion-weighted imaging: diagnosis, prognosis, monitoring

Author	Description	Data Class	Conclusions
Wang et al. [23]	*Study Description* Retrospective, single center *Patient Population* 42 histologically confirmed supratentorial glioma patients *Treatment Regimen* All patients underwent conventional MRI and intravoxel incoherent motion (IVIM) MRI. Parameters between LGG and HGG were compared between different IDH1 mutant statuses using an unpaired t-test. ROC analysis was used to evaluate the diagnostic accuracy of significantly different parameters	Class III	*Results* While IVIM parameters of tumor lesions differed between LGGs and HGG, there was no significant difference in the contralateral normal white matter. ADC values for both LGGs and HGGs were higher for mutated IDH1 when compared with wildtypes. In LGGs, ADC with a cut-off value of 1.180 was able to differentiate between IDH1 mutant and wildtypes with a SEN of 92.86% and a SPE of 80%. In HGGs, ADC with a cut off value of 0.955 was able to differentiate IDH1 mutant and wildtypes with a SEN of 88.89 and a SPE of 71.43%. D* and f values were higher in wild-type IDH1 as compared to IDH1 mutated tumors in HGGs *Author’s Conclusions* Glioma grade and IDH1 mutational status can be predicted using IVIM by simultaneously providing diffusion and perfusion parameters *Comments and Conclusions* The retrospective nature of the data acquisition provides class III data
Mihailovic et al. [9]	*Study Description* Retrospective, single institution *Patient Population* 31 suspected supratentorial glioma patients *Treatment Regimen* All patients underwent surgery and histopathological exam following MRI examination. DWI was acquired for all patients. The uses of ADC maps and parameters such as (∆ADC) and kurtosis in characterizing gliomas was evaluated. T-test and ANOVA was used to compare differences among parameters for each grade	Class III	*Results* ADC values were significantly different between grades II and III (*p* < 0.01) and between grades II and IV (*p* < 0.02) tumors but not between grades III and IV. ∆ADC and kurtosis values were significantly different between grades II and II, grades III and IV, but not between grades II and IV gliomas. ROC analysis revealed that the ∆ADC value had the largest AUC in differentiating between grades II and III glioma (SEN 78%, SPE 89%) at a cutoff value of 0.1·10–3 mm^2^/s *Author’s Conclusions* Parameters derived from ADC maps can be used for overall grading of tumors. ∆ADC value showed the highest SEN and SPE in differentiating grades III and IV tumors but had similar ability as mean ADC in differentiating grade II and III. Parameters that evaluate heterogeneity of ADC values such as the ∆ADC value and kurtosis further improve classification *Comments and Conclusions* The retrospective nature of the data acquisition provides class III data
Hino et al. [25]	*Study Description* Retrospective, single-institutional *Patient Population* 10 LGG patients and 21 HGG patients who underwent IVIM imaging from February 2013 to April 2015. Diagnoses was made histologically through operation or biopsy in all patients *Treatment Regimen* The efficacy of intravoxel coherent motion (IVIM)-derived parameters with three b values and those calculated with multiple b values in differentiating LGG and HGG was studied. The f-max and D-min measured with IVIM derived parameters with 3 b values were compared to those derived from multiple b-values using a paired t-test. F-max and D-min between the LGG and HGG groups were compared using an unpaired t-test	Class III	*Results* F-max value obtained with both the multiple and the three b-values was significantly higher in the HGG than in the LGG group (*p* < 0.0001) F-max calculated with three b-values showed good agreement with the f-max calculated with multiple b-values, although the f-max values obtained with three b-valued was smaller (12.8 ± 5.9%) vs (17.3 ± 7.5%, *p* < 0.0001). (*p* < 0.0001). D-min value calculated with three b-values was not significantly different from that obtained with 13 b values *Author Conclusions* F-max calculated with 3 b-values showed good agreement with f-max calculated with 13 b-values. The possible errors of f-values calculated using the minimum number of b-values is within the acceptable range for clinical use *Comments and Conclusions* The retrospective nature of the data acquisition provides class III data
Chen et al. [42]	*Study Description* Retrospective, single institution *Patient Population* 28 histologically proven LGG patients between 2010 and 2015 who received diagnostic MR with inclusion of DWI *Treatment Regimen* The role of ADC in the assessment of tumor progression in LGG patients was studies. MR images were analyzed separately by 2 neuroradiologists blinded to the patient outcome and classified as stable or progressed in comparison with prior MR imaging using Fisher exact tests and Student t tests	Class III	*Results* Average of ADC_10_ values was lower in patients with tumor progression compared with patients with stable tumors (1.21 ± 0.24 vs 1.49 ± 0.37, *p* = 0.03). SEN, SPE, and overall diagnostic accuracy for ADC_mean_ at a threshold of 1.8 were 84.6, 40, and 62.3%, respectively. ADC10 interval change was able to correctly estimate prediction in 12/13 patients before the lesion progressed on conventional imaging. Averages of ADC_mean_ were not significantly different in patients with progression vs those with stable tumors *Author’s Conclusions* ADC10 interval change is more effective than single time-point quantitative ADC values. Using ADC10 can allow for earlier indication of tumor progression, but these findings need to be confirmed in a larger study *Comments and Conclusions* The retrospective nature of the data acquisition provides class III data
Villaneuva-Meyer et al. [37]	*Study Description* Retrospective, single institution *Patient Population* 100 histopathologic ally confirmed grade II diffuse glioma patients who underwent initial surgery between 2010 and 2014. All patients underwent preoperative MRI including axial DWI, SWI, and contrast enhanced 3D SPGR T1 imaging *Treatment Regimen* All tissue samples from surgery were tested for IDH mutation as well as for loss of heterozygosity in chromosomes 1p and 19q. Purpose was to evaluate MRI markers predictive of IDH status and to use MRI features and IDH status to predict outcomes. Differences in features between IDH wildtype and mutant were analyzed using chi-squared and Mann–Whitney U tests. Cox PH models were created using IDH and MRI characteristics to evaluate the impact of these parameters on clinical outcomes	Class III	*Results* > 45 years of age, multifocal tumor, brainstem location, low ADCmin, mean and max were found to be independent predictors of IDH wildtype status with ADC min having the greatest AUC (0.905, *p* < 0.001). IDH Wildtype status conferred a HR of 6.14 (*p* < 0.001) for death. Median survival was significantly shorter for tumors without 1p19q co-deletion (*p* = 0.009) *Author’s Conclusions* IDH-wildtype grade II is associated with lower ADC and poor clinical outcomes. Combining IDH mutational status with ADC metrics may provide a more accurate predictor of survival. ADCmin was the most predictive of IDH-wildtype status *Comments and Conclusions* The retrospective nature of the data acquisition provides class III data
Falk Delgado et al. [19]	*Study Description* Systematic review involving prospective and retrospective studies *Patient Population* 10 studies involving 430 patients. Studies were eligible if DKI was performed in pathologically confirmed tumors of glial cell origin *Treatment Regimen* Selected studies were assessed for congruency. Extracted data included MK stratified for glioma grade and subtype, diagnostic test accuracy, and DKI sequence parameters. Mean and standard deviation for MK were calculated for each glioma grade. Cross tabulation was performed to describe rate of true-positive, true-negative, false-positive, and false-negative findings	Class III	*Results* There was a mean difference of 0.17 in MK between LGG and HGG (*p* < 0.001). Mean difference in MK between LGG and HGG was higher in astrocytomas (20–22) and in studies with echo time greater than 100 ms, maximal b values less than or equal to 2500 s/mm^2^, ^a^and in repetition time less than 5000 ms *Author’s Conclusions* DKI has a high accuracy in discriminating LGG and HGG. The implementation of DKI can be useful in the clinical workup of suspected gliomas *Discussion* The retrospective nature of the data acquisition provides class III data
Maximov et al. [16]	*Study Description* Retrospective, single institution *Patient Population* 24 patients with newly diagnosed supratentorial glioma (16 HGG and 8 LGG) All patients underwent MRI screening at the time of treatment *Treatment Regimen* DTI, DKI and NODDI metrics were assessed in their ability to differentiate between different grades of glioma. All diffusion metrics were compared among glioma grades using the Mann–Whitney-Wilcoxon test	Class III	*Results* DKI and NODDI models in particular were able to effectively differentiate glioma grades. All diffusion models were able to differentiate grade II and IV gliomas but had more difficulty differentiating grades III and IV gliomas. Maximal AUC values for differentiating grade II and IV values were derived from AK (SEN: 0.86, SPE: 0.99) and TD (SEN: 0.88, SPE: 0.99) *Author’s Conclusions* DKI and NODDI scalar metrics can effectively differentiate LGG and HGG. These metrics are particularly effective at differentiating grade II versus grade III gliomas as well as grade III vs grade IV gliomas *Comments and Conclusions* The retrospective nature of the data acquisition provides class III data
Han et al. [12]	*Study Description* Retrospective, single institution *Patient Population* 39 consecutive patients diagnosed with supratentorial nonenhancing gliomas *Treatment Regimen* The use of ADC derived from DWI values with high b values was compared to ADC values with low b values in differentiating between HGGs and LGGs. Logistic regression analysis was used to compare significant independent factors for discriminating HGG and LGGs. The value of ADC as predictive factors was assessed using log-likelihood criteria	Class III	*Results* ADC values of HGGS were significantly lower than those of LGGs, this difference was greater when ADC values were obtained with high b-values compared to standard b-values. The SEN, SPE, and accuracy for ADC_MEAN_ using high b value were 100, 92.3, and 94.7%, respectively, while the corresponding values using a low b value were 100, 80.8, and 87.2%, respectively *Author’s Conclusions* ADC values obtained by using DWI and a high b-value can be useful in distinguishing HGG and LGG. The greatest likelihood of discriminating HGG and LGG was obtained using a high b-value on discriminant analysis *Comments and Conclusions* The retrospective nature of the data acquisition provides class III data
Cihangiroglu et al. [13]	*Study Description* Prospective, singe center *Patient Population* 53 histologically confirmed patients with a supratentorial glioma were included *Treatment Regimen* All patients underwent DW-MRI. ADC maps acquired using high and low b values were acquired for each patient. The ability of diffusion-weighted MRI using a high b-value and DW-MRI using standard b-value was compared in their ability to preoperatively grade supratentorial glioma. Kruskal–Wallis tests were used to detect significant differences of ADC-related parameters between all tumor grades	Class III	*Results* All ADC values decreased as tumor grade increased for both b values. There were no significant differences in ADC-derived parameters between grade II and III gliomas. Differences in ADCmean, max, and diff were significantly different between grade II and IV gliomas only when using high b-value (*p* < 0.001 for all) *Author’s Conclusions* ADC derived parameters acquired from high b-value DW MRI might provide additional information in prediction of supratentorial glioma grade. Further studies involving a more homogenous patient population are necessary to validate the usefulness of higher b-value DWI-MRI in preoperative grading *Comments and Conclusions* No meaningful contemporaneous or historical control cohort was provided. Therefore, this is class III data
Bai et al. [17]	*Study Description* Prospective, single institution *Patient Population* 69 patients with pathologically proven glioma who underwent both diffusion-weighted imaging and diffusion kurtosis imaging prior to contrast injection *Treatment Regimen* DWI and diffusion kurtosis imaging were compared in their ability to differentiate between high- and low-grade gliomas in terms of diffusivity, fractional anisotropy and mean kurtosis using Mann–Whitney *U* tests	Class III	*Results* Water molecular diffusion heterogeneity index and mean kurtosis displayed greater diagnostic properties than conventional diffusion parameters. Water molecular diffusion heterogeneity index and mean kurtosis had significantly higher heterogeneity indices (0.993 and 0.991, respectively) than ADC, mean diffusivity, and fractional anisotropy (0.886, 0.722, 0.500) in differentiating low- and high-grade gliomas. The AUC for α water molecular diffusion heterogeneity and mean kurtosis were significantly greater than that of apparent diffusion coefficient, mean diffusivity and FA fractional anisotropy in grading gliomas (*p* < 0.05) *Author’s Conclusions* MK mean kurtosis and α water molecule heterogeneity index may serve as an optimal diffusion parameter for grading gliomas in clinic. Both α water molecular diffusion heterogeneity index and MK mean kurtosis were better at grading gliomas than conventional diffusion parameters *Comments and Conclusions* No meaningful contemporaneous or historical control cohort was provided. Therefore, this is class III data
Castellano et al. [43]	*Study Description* Retrospective, single institution *Patient Population* 21 LGG patients *Treatment Regimen* All patients underwent conventional MR and DTI-imaging. The ability of DTI-based histogram and fDM analyses to assess response to TMZ chemotherapy was compared to that of traditional MRI. MR and DTI studies were acquired at baseline, after 3 cycles, and after 6 cycles of TMZ. Group differences of histogram values were analyzed using the Kruskal–Wallis test	Class III	*Results* DTI-histogram parameters showed strong correlations with volume changes, at time preceding tumor size changes on conventional MRI. Changes were more significant on p, MD, and FA than on q maps. In patients with stable disease, the median percentage of voxels with reduction of p an MD values following 3 TMZ cycles was 11.2 and 10.4%, respectively. In patients with progressive disease, the percentage reductions were 31.3 and 27.8%, respectively. The best early discriminant parameter of final volumetric tumor change was percentage change p of histogram 25th percentile values (AUC = 0.889, *p* < 0.0001), where reduction was significantly greater in patients with minor response compared to stable patients. The diagnostic performance of these markers was superior to the percentage change of FLAIR tumor volume (AUC = 0.778, *p* = 0.022) *Author’s Conclusions* DTI-based histogram analysis is an effective tool in detecting and quantifying early tissue changes in LGG following chemotherapy *Comments and Conclusions* The retrospective nature of the data acquisition provides class III data
Hu et al. [22]	*Study Description* Retrospective, single site *Patient Population* 42 newly diagnosed glioma patients between September 2013 and May 2014 *Treatment Regimen* The use of IVIM-derive parameters in grading gliomas preoperatively was studied. Additionally, the parameters ADC, (D), (D*), and f were studied to determine cutoff values for these parameters in differentiating HGG and LGG. ROC analyses were used to determine optimal threshold as well as SEN and SPE for grading	Class III	*Results* The AUC, sensitivity, specificity and the cutoff value, respectively, for differentiating low- from high-grade gliomas for ADC, D and f, and differentiating high- from low-grade gliomas for D* were as follows: ADC, 0.926, 100%, 82.8%, and 0.7 × 10 − 3 mm2/sec; D, 0.942, 92.3%, 86.2%, and 0.623 × 10 − 3 mm^2^/sec; f, 0.902, 92.3%, 86.2%, and 35.3%; D*, 0.798, 79.3%, 84.6%, and 0.303 × 10 − 3 mm^2^/sec *Author’s Conclusions* IVIM-derived parameters were effective in distinguishing HGG and LGG. Significant differences were found in ADC, D, D*, and f between HGG and LGG, indicating their potential use as non-invasive predictors for glioma grade *Comments and Conclusions* The retrospective nature of the data acquisition provides class III data
Server et al. [15]	*Study Description* Prospective, single institution *Patient Population* 78 newly diagnosed brain tumor patients scanned one day prior to surgical excision. All patients received T1 IR MRI and DTI *Treatment Regimen* The ability of intratumoral DTI metrics such as ADC, MD, and FA to differentiate between HGG and LGG was assessed. T tests and Mann–Whitney rank sum test were used to make comparisons between groups	Class III	*Results* ADC, RD, And AD were effectively able to differentiate between LGG and HGG. ADCt, ADt, RDt were all strongly correlated with tumor grade (*p* < 0.0001 for all). Differences in ADC, AD, and RD tumor values and ADC and RD tumor ratios were statistically significant between grades II and III, II and IV and grades II and III-IV. SE and SPE of ADCt with a cutoff value of 1.240 at differentiating between grades II and IV glioma were 100 and 94.4%, respectively *Author’s Conclusions* ADC, RD, and AD are useful parameters derived from DTI that can help with the differentiation of LGG and HGG *Comments and Conclusions* No meaningful contemporaneous or historical control cohort was provided. Therefore, this is class III data
Bisdas et al. [26]	*Study Description* Prospective, single center *Patient Population* 22 consecutive glioma patients. Patients with contraindications to MR imaging (claustrophobia, pacemaker) were excluded *Treatment Regimen* All patients underwent conventional DW-MRI imaging of brain tumors prior to treatment. Exponential ADC images including b values were generated. Parameter values ADC, D, D* and f were tested for differences between tumor sites and contralateral healthy white matter using Welch’s t test or Mann–Whitney U test. Inter-rater agreement between these parameters was assessed by the Kappa value	Class III	*Results* The relative standard deviations were lower in the healthy parenchyma than in the tumor sites (*p* = 0.03). D* (*p* = 0.001) and f(*p* = 0.02) demonstrated significant differences between low- and high-grade gliomas. D, D*, and f in HGG demonstrated significant differences compared to the healthy white matter (*p* < 0.001 < 0.004). Inter-rater agreement for D, D*, and f was generally good with Kappa values of 0.70, 0.67, and 0.83, respectively *Author’s Conclusions* D* estimates did not differ significantly between normal tissue and LGG while f values were slightly higher in healthy white matter than LGG. This is potentially due to the unavoidable inclusion of microscopical capillaries and CSF spaces in the ROI analysis, making the choice of ROI crucial. The inadequate choice of applied b values may have a detrimental effect on acquired IVIM maps, acquiring 30 or more b values in the assessment of IVIM parameters may be optimal. Further studies should explore whether the estimation of IVIM metrics might me more appropriate for follow-up imaging and therapy monitoring of brain tumors *Comments and Conclusions* No meaningful contemporaneous or historical control cohort was provided. Therefore, this is class III data
Cui et al. [11]	*Study Description* Retrospective, single institution *Patient Population* 82 histologically confirmed primary supratentorial glioma patients. Patients with prior needle biopsies or non-microsurgical therapy were excluded *Treatment Regimen* All patients underwent diffusion-weighted MRI. ROC analysis was performed for mean ADC and prognostic factors for glioma pathology. Factors that influence ADC values were examined. Multiple linear regression analysis was used to test associations	Class III	*Results* Mean ADC values of grade II gliomas were significantly higher than those of grade IV gliomas (*p* < 0.05). There was no significant difference in mean ADC values between grade II and III gliomas. In the grade II glioma group, there was a significantly lower ADC in the 1p/19q codeletion subgroup compared to the subgroup without the deletion (1.329 vs 1.711, *p* = 0.004) Multiple linear regression analysis indicated that grade, tumor classification, and 1p/19q status were all significantly correlated with ADC values *Author’s Conclusions* Mean ADC can be used to evaluate prognostic biomarkers in supratentorial glioma. Lower ADC values indicate a more favorable prognosis in LGGs *Comments and Conclusions* The retrospective nature of the data acquisition provides class III data
Balos et al. [10]	*Study Description* Retrospective, single institution *Patient Population* 47 patients with solid brain lesions (25 non neoplastic, 14 low grade, 8 anaplastic glial tumors) *Treatment Regimen* H-MRS and D-MRI was performed in all patients. The Cho/Cr, NAA/Cr and Min/Cr ratios were assessed in their ability to differentiate grade II and grade III gliomas as well as their ability to differentiate gliomas from non-neoplastic mimics. Unpaired two-tailed t tests were used for statistical comparisons between the H-MRS metabolite ratios and ADC values between the groups	Class III	*Results* Cho/Cr ratio was significantly higher in grade II tumors (2.08 vs 1.21, *p* = 0.008) and grade III tumors (2.47 vs 1.21, *p* = 0.001) compared to non-neoplastic legions. NAA/Cr and Min/Cr ratios could not differentiate between non-neoplastic, grade II or grade III tumors. ADC values were significantly different between grade III and grade III glial tumors (*p* = 0.023) *Author’s Conclusions* H-MRS and DWI can aid in the in vivo characterization of foal brain lesions. Metabolic ratios derived from H-MRS are helpful in grading tumors and distinguishing them from non-neoplastic lesions *Comments and Conclusions* The retrospective nature of the data acquisition provides class III data

*MRI* magnetic resonance imaging, *IVIM* intravoxel incoherent motion, *LGG* low-grade glioma, *HGG* high-grade glioma, *IDH* isocitrate dehydrogenase, *ROC* receiver operating characteristics, *ADC* apparent diffusion coefficient, *SEN* sensitivity, *SPE* specificity, D* fast diffusion coefficient, *f* fraction of fast ADC, *DWI* diffusion-weighted imaging, *ANOVA* analysis of variance, *b value* measurement of the degree of diffusion weighting applied, *AUC* area under the curve, *ADC10* tenth percentile histogram cutoff value of ADC, *SWI* susceptibility-weighted imaging, *SPGR* spoiled gradient-recalled echo, *PH* proportional hazards model, *DKI* diffusional kurtosis imaging, *MK* mean Kurtosis, *DTI* diffusion tensor imaging, *NODDI* Neurite orientation dispersion and density imaging, *AUC* area under the curve, *AK* axial kurtosis, *TD* intra-axonal volume fraction, *fDM* functional diffusion maps, *TMZ* temozolomide, *MD* mean diffusivity, *p* pure isotropic component of diffusion, *q* pure anisotropic component of diffusion, *FA* fractional anisotropy, *IR* inversion recovery, *FLAIR* fluid-attenuated Inversion Recovery:, *RD* radial diffusivity, *AD* axial diffusivity, *ADCt* average ADC value of tumor, *ADt* average AD value of tumor, *RDt* average RD value of tumor, *Cho* choline, *Cr* creatinine, *NAA* N-acetyl aspartate, *Min* myoinositol, *H-MRS* proton magnetic resonance spectroscopy

#### Results of individual studies, discussion of study limitations and risk of bias

Although diffusion imaging including ADC mapping is acquired as a part of standard MRI exam for brain imaging, its role is often limited to evaluation for areas of post-surgical cytotoxic edema, effect of anti-angiogenic treatments or assessment for incidental infarcts. Quantitative assessment of diffusion MRI (ADC histogram analysis, ADC mapping, mean diffusivity measurements), intravoxel incoherent motion (IVIM), high b-value diffusion imaging, diffusion tensor imaging (DTI) and diffusion kurtosis imaging (DKI) are considered as advanced diffusion MRI techniques for development of these recommendations.

*Diffusion Imaging For Glioma Grading:* Mihailovic et al. assessed the role of ADC, ∆ADC and K (kurtosis) in glioma grading and found that all three parameters could differentiate between grade II and grade III gliomas with sensitivity of 78, 78, and 57% respectively, and specificity of 78, 89, and 78% respectively [10]. Another study by Balos et al., demonstrated that ∆ADC/ADC difference between grade II and grade III gliomas reached statistical significance (*p* = 0.023) [11]. Another study by Cui et al. found significant differences in grade II versus grade IV gliomas based on ADC and nADC (normalized ADC), but not between grade II and grade III tumors [12]. Two studies compared standard b-value (1000 s/mm^2^) and high b-value (3000 s/mm^2^) ADC maps for pre-surgical grading of supratentorial gliomas [13, 14]. Han et al. found that in non-enhancing supratentorial gliomas, ADC_MIN_, ADC_MAX_, and ADC_MEAN_ values for the non-enhancing HGGs (grade III + IV) were lower than those for LGGs and these differences were more pronounced when using high b-value imaging [13]. In another study with ADC measurements, there were significant differences between grade II and grade IV tumors with standard as well as high b-value diffusion techniques, however, no differences were identified between grade II and grade III gliomas [14]. Naveed et al. explored the utility of advanced MR imaging in grading of oligodendroglial tumors to find no statistical difference between grade II and grade III lesion based on ADC [15]. Server et al. found that the differences in ADC, axial diffusivity and radial diffusivity were statistically significant between grade II and III/IV tumors with AUC of 0.98 [16]. Maximov et al. assessed the ability of DTI, DKI and NODDI (neurite orientation dispersion and density imaging) scalar metrics can be used to differentiate between grade II and grade III/IV gliomas AUCs between 0.82 and 1 [17]. Bai et al. found that water molecular diffusion heterogeneity index and mean kurtosis (AUC of 0.99) offered improved differentiation than mean diffusivity and fractional anisotropy (AUC = 0.5–0.7) in the differentiation of low-grade and high-grade gliomas [18]. In another study, Cao et al. demonstrated no significant difference in ADC values of tumor and peritumoral regions in low-grade and high-grade gliomas [19].

A metanalysis of 10 studies using DKI technique with 430 patients including primary and recurrent gliomas found AUC of 0.94 for discrimination of HGG from LGG, with 0.85 (95% CI 0.74, 0.92) sensitivity and 0.92 (95% CI 0.81, 0.96) specificity with heterogeneity driven by neuropathologic subtype and DKI technique [20]. Intravoxel incoherent motion (IVIM) is a method for simultaneous measurement of diffusion and perfusion [21, 22]. Five studies using IVIM were included in these guidelines [23–27]. Hu et al. assessed the relationship between IVIM derived parameters (ADC, slow diffusion coefficient (D), fast diffusion coefficient (D*), and fraction of fast ADC/perfusion fraction (f)) and the glioma grades. They found that ADC, D, and f were higher in the low-grade gliomas, whereas D* tended to be lower (all *p* < 0.05). The AUC, sensitivity, specificity and the cutoff value, respectively, for differentiating low- from high-grade gliomas for ADC, D and f, and differentiating high- from low-grade gliomas for D* were: ADC, 0.926, 100%, 82.8%, and 0.7 × 10^−3^ mm^2^/sec; D, 0.942, 92.3%, 86.2%, and 0.623 × 10^−3^ mm^2^/sec; f, 0.902, 92.3, 86.2, and 35.3%; D*, 0.798, 79.3%, 84.6%, and 0.303 × 10^−3^ mm^2^/sec [23]. Togao et al. found that ADC and D were higher and f was lower in LGGs as compared to HGGs, while D* showed no separation [25]. In another study, Wang et al. found that ADC and D were higher and D* and f were lower in LGGs as compared to HGGs. The area under the receiver operating characteristic (ROC) curve (AUC) was 0.937, 0.898, 0.770, and 0.838, respectively for ADC, D, D* and f [24].

Several additional multiparametric studies combine diffusion imaging with other advanced imaging techniques. Caulo et al. found that a cut off of > 2.61 for restricted diffusivity was able to distinguish between low and high grade gliomas with an AUC of 0.88 [28]. Tietze et al. found poor to fair correlation between presurgical MRI with perfusion and diffusion imaging, and MET-PET scans in 13 patients with gliomas where MET-PET was considered a surrogate for tumor heterogeneity and invasion [29]. Perez et al. assessed the performance of perfusion and diffusion imaging in differentiating between high-grade and low-grade gliomas to find that all perfusion parameters and minimum ADC were able to differentiate between all glioma grades [30]. Lin et al. found that IDH-mutant and 1p/19q co-deleted oligodendrogliomas can be stratified by grades using advanced magnetic resonance imaging techniques including DWI and DSC perfusion imaging [31]. In a study of 49 newly diagnosed gliomas, Sakata et al. found that minimum ADC, tumor to normal 18-F FDG uptake and mean amide proton transfer were able to differentiate between grade II and grade III/IV tumors with AUC of 0.78, 0.84 and 0.72, respectively [32]. In another multiparametric approach, Durmo et al. found that normalized ADC, normalized CBF and normalized CBV were able to differentiate between low and high-grade gliomas, moreover when all the parameters were combined, the sensitivity and specificity reached 100% [33]. Liu et al. compared the performance of 3D pseudocontinuous ASL with DWI for differentiating low-grade from high-grade diffuse gliomas and found that the ADCmean (AUC 0.81) and ADC min (AUC 0.85) outperformed CBFmax, rCBFmean and rCBFmax (AUCs of 0.70–0.74) [34]. Yoon et al. used a multiparametric approach with ADC, rCBV, and MRS and found that using a cut-off value of 0.85, only ADC was able to separate low and high grade gliomas with an AUC of 0.76 [35].

In a study with 32 patients, Fudaba et al. found that when minimum ADC and choline/creatine ratios were used together, low grade gliomas could be differentiated from high grade gliomas with sensitivity and specificity of 87 and 88.9%, respectively [36]. Jeong et al. utilized isotropic diffusion spectrum imaging (IDSI) and compared it with AMT-PET uptake regions and glioma proliferative index. They found that IDSI-derived cellularity showed significant correlation with glioma proliferative index (based on ki-67 labeling) with R = 0.95 and *p* < 0.001 [37].

*Diffusion Imaging for Genomics and Prognostication:* In a study with 35 grade II gliomas, Cui et al. found that a threshold mean ADC value of 1565 × 10^–6^ mm^2^ /s could predict the 1p/19q chromosomal status with 72% sensitivity and 88% specificity (AUC 0.82, 95% confidence interval 0.68–0.97) and could distinguish low-grade glioma with low-risk factors from the high-risk group (*p* < 0.01). The mean ADC value could be used as a non-invasive tool to evaluate the prognosis with lower ADC values indicative of a favorable prognosis in LGGs [11]. In another study with 100 grade II gliomas, IDH wild-type tumors were associated with a lower ADC. ADC_min_ threshold of 0.9 × 10^–3^ mm^2^/s or less provided the greatest sensitivity and specificity (91 and 76%, respectively) in defining IDH wild-type grade II diffuse gliomas. Combining low ADC_min_ with IDH wild-type status conferred worse outcomes than did IDH wild-type status alone [38]. On the other hand a study with 31 LGGs by Leu et al. did not find any significant difference between IDH-WT, IDH-Mutant/1p19q^+^ and IDH-mutant/1p19q^−^ gliomas using ADC or rCBVleu [39].

*Diffusion Imaging for Post-treatment monitoring:* Neill et al. performed diffusion imaging in 122 patients with baseline diagnosis of grade II gliomas, who presented with recurrence (41% Grade II, 43% Grade III and 15% Grade IV). The relationship between various nADC and nFA, and progression-free survival (PFS) till subsequent re-recurrence was tested after adjusting for age, EOR and subsequent treatment and found to be significant. nADC values were lower and nFA values were higher in patients with poorer PFS [40]. Lotumolo et al. assessed for lower ADC values within the tumors as compared to prior MRI scans. They found that DWI imaging had accuracy of 87.5% for differentiation between disease progression and response with RANO criteria as gold standard [41]. In a smaller study with 12 patients, Rossi-Espagnet et al. evaluated the ability of ADC, rCBV and 18F-FDOPA PET to predict stable or progressive disease at 1-year follow-up and found only 18F-FDOPA to be useful [42]. In another retrospective study of 28 LGGs, the interval change of ADC values between sequential scans was used to differentiate stable disease from progression. The authors found that the interval change of ADC_10_ values can be used to identify progression versus stability of low-grade gliomas with a diagnostic accuracy of 86% and on an average 8 months before apparent radiologic progression on conventional MR imaging [43]. In another study of 21 LGGs, Castellano et al. demonstrated the utility of DTI in early detection of mid-therapy response, well before changes were seen on conventional imaging. Early changes in DTI correlated well with symptomatic seizure control as well as with final tumor volume change [44].

#### Synthesis

Since our prior recommendations were published, there has been a significant amount of work published on role of diffusion imaging in the assessment of low-grade gliomas, however all the papers have produced class III evidence. The ability to differentiate between LGG and HGG is confirmed by 28 articles. As such, there is no rationale to alter the previous level II recommendation that the addition of diffusion weighted MR imaging could be used in the assessment of suspected low-grade gliomas, for the purposes of discriminating between tumor subtypes and detection of higher-grade diagnoses. Also, diffusion imaging is considered part of standard MR imaging and falls under the Level II recommendations of MRI which remain unaltered.

The evidence supporting the role of diffusion imaging in genomics, prognosis and post-treatment monitoring is not as robust as for grading, again with all evidence falling in the class III category. Given the class III evidence, a level III recommendation for diffusion imaging for identifying tumor genomics, prognosis and post treatment monitoring in WHO Grade II diffuse glioma is warranted.

### Perfusion imaging

#### Study selection and characteristics

On full text-review, 14 articles concerning various perfusion techniques, all providing class III evidence were eligible for use in guideline creation as outlined in Table 4. Another 18 articles on multi-parametric MR imaging studies including perfusion imaging as one of the techniques are included in separate table on multi-parametric MR imaging (Table 6). Three articles concerning multi-modality imaging where MR perfusion imaging was investigated along with amino acid PET are included in Table 7. Thus, a total of 31 full text articles, all providing class III evidence concerning perfusion MR were included for guideline creation.

**Table 4:** Perfusion-weighted imaging (including DSC, DCE and ASL): diagnosis, prognosis, monitoring

Author	Description	Data Class	Conclusions
Liu et al. [56]	*Study Description* Retrospective, single institution study *Patient Population* 31 HGG and 11 LGG patients with who had undergone initial MRI under tumor protocol from October 2006 to October 2012. All patients had histopathological diagnosis of astrocytotic tumor without oligodendroglial components along with T2* DSC-PWI-MRI along with MR imaging *Treatment Regimen* The ability of Ktrans and vp derived from T2* DSC-PW-MRI to differentiate between glioma grades was studied. Mann–Whitney U tests were performed to compare histogram parameters among different grades of glioma. Associations between Ktrans and Vp and glioma grade was assessed using Spearman correlation coefficients	Class III	*Results* Ktrans and Vp values were significantly correlated with tumor grade. Mean Ktrans was 3.79 in HGG and 1.45 in LGG (*p* < 0.0001). Mode of Vp was 2.15 in HGG while it was 0.6 in LGG (*p* < 0.0001). While Ktrans and Vp were able to differentiate Grade II and IV gliomas, there were no significant differences in these parameters between Grade III and IV gliomas. Mean Ktrans had the highest AUC with a cutoff value of 1.92 in differentiating HGG and LGG with a SEN of 100% and SPE of 85.71%). There were no significant correlations between Ktrans and Vp measurements *Author’s Conclusions* Pharmacokinetic parameters fromT2* DSC-PW-MRI-based histogram is useful for glioma grading. Ktrans from T2* DSC-PW-MRI-based histogram was the most significant parameter in differentiating HGG and LGG based on AUC. The lack of correlation between Ktrans and Vp measurements suggests different processes in pathologic processes in tumor angiogenesis in terms of CBV versus microvascular permeability *Comments and Conclusions* The retrospective nature of the data acquisition provides class III data
Komatsu et al. [52]	*Study Description* Retrospective, single institution *Patient Population* 102 pathologically confirmed glioma patients from June 2010 to June 2014. All patients underwent MRI with ASL *Treatment Regimen* The effectiveness of ASL (arterial spin labeling) in grading gliomas was studied. Differences in TBF derived from ASL among high-and low-grade glioma groups were assessed using the Mann–Whitney U test	Class III	*Results* TBF tended to increase with tumor grade. tVI (TBF/cerebral blood flow) was significantly higher in HGG than in LGG (1.46 vs 1.05, *p* = 0.003). SEN and SPE of ASL for differentiating between LGG and HGG was 62.7 and 72.5%, respectively. tVI of grade II (1.05) and grade IV gliomas (1.46) was significantly different, but no differences were observed in tVI between grades II and II or grades III and IV gliomas *Author’s Conclusions* The ASL method was effective in differentiating HGG and LGG. Given that it does not require contrast and only takes 3 min longer than standard MRI, it may be a beneficial addition to MRI protocol for grading *Comments and Conclusions* The retrospective nature of the data acquisition provides class III data
Brendle et al. [59]	*Study Description* Retrospective, single-institution analysis *Patient Population* 40 brain tumor patients with complete DCE and ASL perfusion datasets and histologically confirmed brain tumors between November 2012 and August 2015 *Treatment Regimen* Tumors were classified as either astrocytic tumor with IDH wildtype, astrocytic tumor with IDH mutation, or oligodendroglia tumor using immunohistochemistry. The role of DCE and ASL perfusion parameters in differentiating glioma grade as well as IDH and ATRX mutation status was evaluated	Class III	*Results* Ve from DCE perfusion appears to be effective in discriminating between high- and low-grade gliomas (SE*N* = 1, SPE = 0.8, *p* = 0.024) while ASL perfusions was able to differentiate between IDH mutants and wildtypes (SE = 0.014, SP = 0,88, *p* = 0.014) *Author Conclusions* DCE and ASL perfusion parameters can provide complementary information in perfusion imaging perfusion parameters can discriminate LGG and HGG and ASL perfusion parameters can differentiate IDH and ATRX mutation status *Comments and Conclusions* The retrospective nature of the data acquisition provides class III data
McCullough et al. [61]	*Study Description* Retrospective, single institution *Patient Population* 146 newly diagnosed diffuse grades II-IV infiltrating glioma patients between January 2007 and December 2014. All patients underwent had a DSC perfusion sequence prior to resection and histopathological analysis *Treatment Regimen* 146 patients were retrospectively assessed. The role of rCBV alongside histopathological analysis as a predictor of survival in patients with surgically treated gliomas was evaluated. KM curves and a Cox PH model was used to evaluate the effect of rCBV value and histological grading on overall survival	Class III	*Result* rCBV analysis was able to predict overall survival, as it performed similarly in predicting survival to WHO grading. rCBV was strongly correlated with histopathological grade (*p* < 0.0001). Patients with grade III pathology had a HR of 3.91 on the Cox-PH for overall survival compared to patients with grade III pathology (*p* = 0.018). An increase of 1.0 in rCBV had a HR of 1.12 (*p* = 0.002) *Author’s Conclusions* Preoperative rCBV analysis can predict overall survival and was comparable to WHO grade in stratifying risk *Comments and Conclusions* The retrospective nature of the data acquisition provides class III data
Zeng et al. [51]	*Study Description* Retrospective, single institution *Patient Population* 58 patients with pathologically confirmed supratentorial glioma who underwent preoperative 3D continuous arterial spin labeling *Treatment Regimen* The effectiveness of CBF maps derived from 3d pCASL in assessing, grade, proliferation, and prognosis of gliomas in terms of overall and progression-free survival was evaluated. 1-way ANOVA was used to compare differences in parameters among multiple groups while independent samples t- tests were used compare differences in parameters among HGG and LGG	Class III	*Results* Gliomas with a higher blood flow were associated with lower overall and progression-free survival. Higher CBFmax and rCBF max were associated with increased malignancy in gliomas while higher maximum relative CBF were associated with better survival in GBM (*p* < 0.01). AUC for CBF and rCBF in differentiating HGG form LGG were 0.828 and 0.863, respectively *Author’s Conclusions* 3d pseudocontinuous arterial spin-labeling-derived CBF maps are effective in the preoperative evaluation of gliomas. IN accordance with prior studies, increased CBFmax and rCBFmax values from pCASL were associated with lower progression-free survival *Comments and Conclusions* The retrospective nature of the data acquisition provides class III data
Arevalo-Perez et al. [57]	*Study Description* Retrospective, single- institution analysis of consecutive patients with grade II and III oligodendrogliomas *Patient Population* 24 consecutive patients with pathologically confirmed oligodendroglioma from January 2011–2015 *Treatment Regimen* Pretreatment DCE-MRI perfusion scans were acquired for each patient. Ability of DCE MRI to differentiate between grade II and III oligodendroglioma was evaluated in terms of V_p_ and K_trans_ using a Mann–Whitney U test	Class III	*Results* Vp_mean_ values (*p* = 0.03) were significantly higher in grade III oligodendrogliomas compared to grade II oligodendrogliomas. Vp_mean_ cut-off of 2.35 provided the best combination of SEN (70%) and SPE (70%) to distinguish between grade II and II oligodendrogliomas *Authors Conclusions* DCE-MRI perfusion parameters outperformed ADC in discriminating HGG and LGG Pretreatment analysis of Vp_mean_ can differentiate between grade II and III oligodendrogliomas *Comments and Conclusions* The retrospective nature of the data acquisition provides class III data
Kang et al. [45]	*Study Description* Prospective, single institution *Patient Population* 70 patients with suspected primary glioma between March 2013 and August 2014 *Treatment Regimen* 30 patients who had undergone both SE-perfusion and VSI at 24–48-h intervals were assigned to group 1, where the optimal cutoff value of VSI and rCBV to distinguish HGG and LGG would be generated. 20 patients assigned to group underwent SE-perfusion and 20 patients assigned to group 3 underwent VSI. The optimal cutoff values established in group 1 were used to predict glioma grading in groups 2 and 3 ANOVA and Least Significant Difference test were used to analyze differences in VSImax and rCBV max in patients with gliomas of different grade	Class III	*Results* VSI and rCBV values of grade III and IV gliomas were significantly higher than those of grade II gliomas (*p* < 0.01). No significant differences in these parameters between grade III and IV gliomas. AT the optimal cutoff of 138.3 um, the sensitivity and specificity of VSImax values were 95.24 and 100%, respectively. At the optimal cutoff values of 5.73, the sensitivity and specificity of rCBV values was 85.71 and 88.9%. Compared with histological diagnosis, predictions of VSI for glioma grade had a 100% accuracy, whereas that of rCBV had an 85% accuracy *Author’s Conclusions* VSI may serve as an effective tool for the diagnosis of patients with gliomas. VSI was more effective at predicting glioma grade than rCBV *Comments and Conclusions* No meaningful contemporaneous or historical control cohort was provided. Therefore, this is class III data
Nguyen et al. [46]	*Study Description* Prospective, single institution *Patient Population* 48 newly diagnosed astrocytoma patients who underwent MR and DSC MRI preoperatively *Treatment Regimen* VP and Ktrans derived from DCE-MR imaging and CBV values derived from DSC-MR imaging were compared in their ability to grade astrocytomas. Differences in these parameters based on grade were assessed using the Kruskal–Wallis and Mann–Whitney U tests. ROC curves were plotted to determine the accuracy of each parameter	Class III	*Results* Vp and Ktrans parameters derived from DCE had the same diagnostic accuracy as rCBV derived from DSC. Median Vp_Φ and *K*^trans^_Φ were lower for grade II compared with grade II astrocytoma (*p* < 0.05), while median rCBV Vp_SI, and *K*^trans^_SI values were not significantly different between glioma grades *Author’s Conclusions* The performance of diagnostic parameters derived from DCE-MR and DSC-MR in differentiating astrocytoma grade is similar *Comments and Conclusions* No meaningful contemporaneous or historical control cohort was provided. Therefore, this is class III data
Nguyen et al. [62]	*Study Description* Prospective, single institution *Patient Population* 46 newly diagnosed glioma patients between December 2008 and March 2011. Patients with prior surgery or biopsy, contrast intolerance, and pregnancy were excluded. All patients with resection followed by histopathologic diagnosis following MR imaging *Treatment Regimen* The prognostic value of Ktrans and vp obtained from DCE MR was assessed. Patients were split into either a high Vp/Ktrans and low Vp/Ktrans group, and KM curves were obtained for each. Cox PH models were used to determine the effects of potential prognostic variables on overall survival	Class III	*Results* Glioma patients with a high Ktrans and vp had significantly lower survival rates than those with low Ktrans and vp. Low Karnofsky (HR: 2.56(score and high contrast transfer coefficient (HR: 4.53) were associated with lower survival rates in a multivariate model. (*p* < 0.05) *Author’s Conclusions* Parameters derived from DCE imaging have potential as a biomarker for prognosis. High transfer coefficients were associated with lower survival rates *Comments and Conclusions* No meaningful contemporaneous or historical control cohort was provided. Therefore, this is class III data
Falk et al. [47]	*Study Description* Prospective, single institution *Patient Population* 25 patients with suspected LGG based on morphological MRI findings were enrolled from May 2010 and November 2012 *Treatment Regimen* All but one patient had the study MRI preoperatively. All patients underwent DCE-MRI and then DSC-MRI 10 min afterwards. The ability of histogram perfusion parameters to distinguish between grade II and III glioma was evaluated. CBV, CBF and K_trans_ from DCE and CBV were included as perfusion parameters while CBF and K_app_ from DSC were included. Student’s t test and Mann–Whitney U tests were used for statistical analysis	Class III	*Results* From DCE, skewness of K_trnas_ was most effectively able to differentiate between grade II and grade III gliomas (AUC 0.76). Skewness of ktrans was 1.60 in in grade II gliomas while it was 2.86 in grade III gliomas (*p* = 0.07). Standard deviation (SD) of rCBF was most effective in differentiating between grade II and II gliomas in DSC (AUC 0.80). SD of rCBF was 173.56 in grade II gliomas while it was 330.51 in grade III gliomas (*p* = 0.02) *Author Conclusions* Histogram analysis of perfusion parameters from DCE and DSC can potentially be used for the grading of suspected LGG. Ktrans from DCE and rCBF from DSC were most effective at differentiating grade *Comments and Conclusions* No meaningful contemporaneous or historical control cohort was provided. Therefore, this is class III data
Jung et al. [49]	*Study Description* Retrospective, single institution *Patient Population* 28 patients with histologically confirmed glioma and initial DCE MR imaging between January 2010 and October 2012 *Treatment Regimen* All patients were examined before surgery or biopsy with conventional MR imaging and T1-weighted dynamic contrast-enhanced perfusion MR imaging. The utility of histogram analysis of pharmacokinetic parameters such as Ktrans, Ve and Vp in the grading of gliomas was studied. ROC curves were generated to determine the cutoff percentile for differentiating HGG and LGG as well as grade 3 and 4 tumors	Class III	*Results* The values of all parameters increased with glioma grade. With a SEN of 91.2% and a SPE of 100%, Ktrans C98 was the most significant parameter in distinguishing HGG form LGG. Ktrans of C98. Ve of C90, and Vp of C84 exhibited the highest AUCs of 0.912, 0.939, and 0.769, respectively, for differentiating HGG and LGG *Author Conclusions* Histogram analysis of pharmacokinetic parameters can be used to effectively grade gliomas. The results were in agreement with previous studies, as Ktrans was the most significant parameter in grading *Comments and Conclusions* The retrospective nature of the data acquisition provides class III data
Kim et al. [44]	*Study Description* Retrospective, single institution *Patient Population* 63 patients with astrocytic tumors who underwent 3 T MRI with dynamic susceptibility contrast PWI from February 2012 to April 2012 *Treatment Regimen* The use of a cumulative (nCBV) histogram for glioma grading was studied, and the best parameter for differentiating glioma grades was identified. The diagnostic accuracies of different percentile values of the cumulative nCBV histogram in differentiating HGG and LGG glioma were compared. Student t-tests or Mann–Whitney U-tests were performed to compare histogram parameters between LGG and HGG. ANOVA or the Kruskal–Wallis test was performed to compare histogram parameters among different glioma grades	Class III	*Results* The 99th percentile nCBV exhibited the highest AUC for differentiating high- and low-grade gliomas (*p* < 0.001) (AUC: 0.893). Mean (*p* = 0.002) and peak height (*p* < 0.001) were also significantly different between grades II and IV gliomas. nCBV C99 cutoff of 4.681 exhibited SEN, SPE, and accuracy of 85.2, 100, and 87.3%, respectively. Diagnostic accuracy of NCBV C99 was significantly higher than that of mean nCBV (*p* = 0.016) *Author’s Conclusions* The cumulative nCBV histogram method can provide high diagnostic accuracy in differentiating glioma grade. nCBV99 had the highest accuracy in differentiating between grades of glioma *Comments and Conclusions* The retrospective nature of the data acquisition provides class III data
Jia et al. [48]	*Study Description* Retrospective, single institution *Patient Population* 65 oligodendroglioma patients *Treatment Regimen* The use of the volume transfer constant (K_trans_) and the extravascular extracellular space per unit volume of tissue (V_e_) in distinguishing low grade and anaplastic oligodendroglioma was studied. Differences in the two parameters between the low-grade and anaplastic groups were compared using the Mann–Whitney rank-sum test. ROC curve analyses were used to determine cut-off values for the K_trans_ and V_e_ that could differentiate between low and anaplastic oligodendrogliomas	Class III	*Results* Values for K_trans_ and V_e_ in low-grade oligodendrogliomas were significantly lower than those in anaplastic oligodendrogliomas (*p* < 0.001 for both). Cut off values of 0.037 min^−1^ for Ktrans and 0.079 for V_e_ could effectively distinguish between low-grade and anaplastic oligodendrogliomas *Author’s Conclusions* DCE-MRI can distinguish differences in microvascular permeability between low-grade and anaplastic oligodendrogliomas *Comments and Conclusions* The retrospective nature of the data acquisition provides class III data

*HGG* high-grade glioma, *LGG* low-grade Glioma, *MRI* magnetic resonance imaging, *DSC* dynamic susceptibility contrast, *PWI* perfusion-weighted imaging, *Ktrans* volume transfer constant, *Vp* plasma volume, *AUC* area under the curve, *SEN* sensitivity, *SPE* specificity, *PW* perfusion weighted, *CBV* cerebral blood volume, *TBF* tumor blood flow, *ASL* arterial spin labeling, *DCE* dynamic contrast enhanced, *IDH* isocitrate dehydrogenase, *ATRX* X-linked nuclear protein, *DSC* dynamic susceptibility contrast, *rCBV* relative cerebral blood volume, *KM* Kaplan Meier, *PH* proportional hazards, *WHO* World Health Organization, *HR* hazard ratio, *CBF* cerebral blood vlow, *pCASL* pseudo-continuous arterial spin labeling, *ANOVA* analysis of variance, *CBF* cerebral blood flow, *rCBF* relative cerebral blood flow, *ADC* apparent diffusion coefficient, *SE* spin echo, *VSI* vessel size imaging, *ROC* receiver operating characteristics, *Vp_Φ* plasma volume obtained from phase-derived vascular input function, *Ktrans_Φ* volume transfer constant obtained from phase-derived vascular input function, *Ktrans_SI* volume transfer constant obtained from magnitude-derived vascular input function, *Kapp* apparent transfer constant, *Ve* extravascular fluid volume, *PWI* perfusion-weighted imaging, *nCBV* normalized cerebral blood volume, *AUC* area under the curve

#### Results of individual studies, discussion of study limitations and risk of bias

*Grading:* Out of the 31 full text articles, 28 articles informed our recommendations on grading with 4 out of these 28 providing additional data on IDH1 mutation assessment. There are 2 articles on survival/prognosis and 3 articles on post treatment follow-up. The three key perfusion techniques that have been used in these articles include dynamic susceptibility contrast (DSC), dynamic contrast enhancement (DCE) and arterial spin labeling (ASL). In a DSC perfusion study, Kim et al. looked at cumulative histogram analysis of normalized CBV and found that 99 percentile CBV values were able to differentiate between LGGs and HGGs [45]. In another study using DSC perfusion imaging, Kang et al. found that rCBV and vessel size imaging values (VSImean) had 85 and 100% accuracy, respectively in differentiating low and high grade gliomas [46]. In a study directly comparing DSC and DCE techniques, Nguyen et al. found that selected parameters from both were useful in differentiating between grade II and grade III gliomas and there was no difference between diagnostic accuracy of the two techniques [47]. A similar study by Falk et al. using histogram analysis found that Ktrans skewness (DCE) and rCBF standard deviation were superior in differentiating between grade II and III gliomas with AUC of 0.76 and 0.80, respectively [48]. In a study of 27 grade II and 38 grade III oligodendrogliomas by Jia et al., cut-off values of the Ktrans (0.037 min^−1^) and Ve (0.079) could be used to distinguish between the two groups (Ktrans: Sensitivity 97.4% Specificity 96.3% and AUC = 0.99; Ve: Sensitivity 94.7% Specificity 100% and AUC = 0.99)[48]. In similar studies using DCE by Jung et al. and Li et al., several parameters were useful in differentiating between low and high grade gliomas [49, 50]. A study by Brendle et al. found that Ktrans, CBF and Ve (from DCE) were able to differentiate between grade II from grade III + IV gliomas, but CBF derived from ASL was not useful. Zeng et al. evaluated the role of 3D ASL in grading and found significant differences in ASL parameters between LGGs and HGGs with AUCs of 0.82 and 0.86 for maximum CBF and maximum relative CBF, respectively [51]. Similar studies by Komatsu et al. and Qu et al. also demonstrated the utility of various ASL parameters to differentiate between low and high grade gliomas [52, 53]. In a multiparametric study with modest sample size, when CBF, CBV and ADC measurements from tumor and peritumoral region were used, LGG and HGG could be discriminated with 100% sensitivity and specificity [33]. Naveed et al. explored the utility of advanced MR imaging in grading of oligodendroglial tumors to find no statistical difference between grade II and grade III lesion based on rCBV [15]. Wang et al. and Saini et al. demonstrated the utility of rCBV in differentiating between grade II and grade III and low and high grade gliomas [54, 55]. Liu et al. used histogram analysis of permeability parameters derived from first-pass pharmacokinetic modeling based on DSC perfusion imaging to differentiate between low and high grade gliomas [34]. Sengupta et al. developed a machine learning framework based on T1 perfusion MRI features that was able to differentiate between grade II and grade gliomas with classification error of 3.7% and AUC ranging from 0.76–0.81 [56].

Caulo et al. used a multiparametric MR approach in 118 gliomas to differentiate grade II from grade III/IV tumors and found that rCBV in contrast-enhanced regions showed AUC of 0.93 at a cutoff 2.59 [27]. Tietze et al. found poor to fair correlation between presurgical MRI with perfusion and diffusion imaging, and MET-PET scans in 13 patients with gliomas where MET-PET was considered a surrogate for tumor heterogeneity and invasion[28]. Perez et al. assessed the performance of perfusion and diffusion imaging in differentiating between high-grade and low-grade gliomas to find that all perfusion parameters and minimum ADC were able to differentiate between all glioma grades[29]. Another study by Perez et al. demonstrated that Vp_mean_, a DCE MRI parameter can differentiate between grade II and grade III oligodendrogliomas with an AUC of 0.757 [57]. Yoon et al. used a rCBV cut-off of 2.44 to differentiate between low- and high-grade gliomas with an AUC of 0.81. In the same study, AUC for ADC, MRS ratios and FDG-PET were lower in comparison to rCBV[34].

Lin et al. found that IDH-mutant and 1p/19q co-deleted oligodendrogliomas can be stratified by grades using advanced magnetic resonance imaging techniques including DWI, SWI and DSC perfusion imaging. The combination of SWI and DSC perfusion resulted in sensitivity and specificity of 100 and 93%, respectively [31]. In another multiparametric approach, Durmo et al. found that normalized ADC, normalized CBF and normalized CBV were able to differentiate between low and high-grade gliomas, moreover when all the parameters were combined, the sensitivity and specificity reached 100% [33]. Fudaba et al. assessed multiple pulsed ASL measurements to separate low from high grade gliomas with sensitivity and specificity values ranging from 0.65–0.80 to 0.66–0.77, respectively [36].

*Genomics, prognostication, and post treatment monitoring:* There is paucity of robust literature on role of perfusion imaging in additional pre-and post-treatment imaging of LGGs. Of the 4 articles on LGG genomic assessment, Leu et al. found negative ability of rCBV to differentiate between IDH and 1p19q mutation categories in grade II gliomas [39]. Brendle et al. demonstrated that CBF by ASL perfusion enabled discrimination of astrocytomas with and without IDH mutation (*p* = 0.014, sensitivity = 0.75, specificity = 0.88), however, grade II, III and IV tumors were included for this analysis [58]. Similarly, Liu et al. demonstrated the ability of ASL to distinguish between IDH mutant and WT gliomas, however, again in a combined cohort of grade II and grade III tumors [34]. Stadlbauer et al. assessed tumor vascularity with quantitative blood oxygen level–dependent imaging and vascular architecture mapping and demonstrated clear differentiation of glioma grades as well as IDH mutant and WT grade II gliomas [57].

There is paucity of adequate literature on role of perfusion imaging in predicting outcomes in WHO Grade II diffuse gliomas. Only two studies were identified in this category, both studies combined all glioma grades for survival analysis [59, 60]. There is very limited evidence on role of perfusion in WHO Grade II diffuse glioma follow-up. In a small sample study (*n* = 12), Rossi-Espagnet et al. found no difference between CBV parameters of patients with subsequent progression or stable disease [42].

#### Synthesis

Similar to diffusion, there is robust body of evidence pertaining to the role of perfusion imaging (DCE, DSC and ASL) in the differentiation glioma grades, however all the papers have produced class III evidence. Out of the 28 studies on DCE and DSC imaging, all studies but 1 were useful in differentiating low from high grade gliomas. Out of 5 articles on ASL, 4 were useful for grade based differentiation. As such, there is no rationale to alter the previous level II recommendation that the addition of perfusion weighted MR imaging could be used in the assessment of suspected low-grade gliomas, for the purposes of discriminating between tumor subtypes and detection of higher-grade diagnoses.

The evidence supporting the role of perfusion imaging in genomics, prognosis and post-treatment monitoring is not as robust as for grading, again with all evidence falling in the class III category. Given the class III evidence, a level III recommendation is made for perfusion imaging for identifying tumor genomics, prognosis and post treatment monitoring in low grade gliomas is made that DSC, DCE and ASL may be considered if this information is not available through other techniques.

### 1H magnetic resonance spectroscopy

#### Study selection and characteristics

On full text-review, 2 articles concerning only MRS techniques, providing class III evidence were eligible as outlined in Table 5. Another 6 articles on multiparametric MR imaging studies including MRS as one of the techniques are included in Table 6. Two articles concerning multi-modality imaging where MRS imaging was investigated along with FDG PET and MET-PET are included in Table 7. Thus, a total of 10 full text articles, all providing class III evidence concerning MRS were included for guideline creation. Out of these 10 articles, 8 articles on grading and/or initial assessment, one article on post-treatment monitoring and a single article on progression free survival prediction informed our recommendations in this category.

**Table 5:** Magnetic resonance spectroscopy: diagnosis, prognosis, monitoring

Author	Description	Data class	Conclusions
Tong et al. [65]	*Study Description* Prospective, single institution *Patient Population* 49 pathologically confirmed glioma patients and 20 normal control subjects *Treatment Regimen* The ability of changes in the Cho/NAA ratio measured by dynamic 1H-MRS to differentiate between HGG and LGG was evaluated. ANOVA and post-hoc Scheffe’s tests was used to measure differences in Cho/NAA in HGG and LGG. ROC curves were used to determine cutoff values with best SPE and SEN	Class III	*Results* Glioma patients regardless of grade had a significantly different Cho/NAA ratio in tumor area on the 0 min scan than on the 6 min scan (p-0.017), with no significant difference in surrounding normal tissue areas between 0 and 6 min (*p* = 0.121). Difference of Cho/NAA ratio between 0 and 6 min was significantly higher in HGG than LGG (3.86 vs 0.81, *p* < 0.001). No significant difference in LGG and control subjects. Differences in Cho/NAA > 1.07 had SE and SPE of 87.5 and 80%, respectively of HGG *Author’s Conclusions* Dynamic H-MRS can be useful for differentiating HGG and LGG and provide insight into the heterogeneity within the tumor *Comments and Conclusions* No meaningful contemporaneous or historical control cohort was provided. Therefore, this is class III data
Raschke et al. [63]	*Study Description* Retrospective, single institution *Patient Population* 24 tumor patients *Treatment Regimen* The use of group average single voxel MRS to estimate normal brain and tumor proportions within each voxel of 1H 2dMRSI from glioma patients was assessed. An LCModel basis set was used to represent normal brain spectra as well as different glioma grades from single voxel spectra and spectra of healthy controls. Simulations were used to evaluate the performance of the LCModel by modeling tissue heterogeneity, noise, and line widths	Class III	*Results* LCModels and whole tissue representation can be used to decompose 1H MRSI spectra to identify tumor extent, infiltration, and overall tumor grade. Infiltrative tumor proportions as low as 20% can be identified at the typical 1H MRS signal-to-noise found in vivo. Accuracy of grading between grade II and IV glioma was 86% with this method *Author’s Conclusions* LCModel and whole tissue representations can be used to decompose 1H MRSI spectra into proportions of normal and abnormal tissue. This can help identify tumor extent, infiltration, and overall tumor grade. This methodology could potentially be applied toother tumor types both in and outside of the brain *Comments and Conclusions* The retrospective nature of the data acquisition provides class III data

*Cho* Choline, *NAA* N-acetyl aspartate, *MRS* magnetic resonance spectroscopy, *HGG* high-grade glioma, *LGG* low-grade glioma, *ANOVA* analysis of variance, *ROC* receiver operating characteristics, *SPE* specificity, *SEN* sensitivity, *1H 2dMRSI* free-induction-decay magnetic resonance spectroscopic imaging

**Table 6:** MR studies with any combination of technique: diagnosis, prognosis, and monitoring

Author	Description	Data Class	Conclusions
Sengupta et al. [58]	*Study Description* Retrospective, single institution *Patient Population* 53 histologically confirmed glioma patients (26 Grade IV, 12 Grade II, 15 Grade II) who underwent conventional MRI and T1 perfusion MRI *Treatment Regimen* The use of T1 perfusion parameters in addition to conventional MRI in differentiating intermediate glioma grades was studied. Tumor subparts were segmented using a method involving a combination of MRI images and T1 perfusion parameters with a SVM classifier. The Tukey–Kramer test was used to compare mean values of each of the features of patients of different grades. An SVM classifier was used to differentiate between intermediate-grade gliomas. The (SBS) wrapper method and random forest were also tried as feature reduction techniques to obtain a feature set to be used in the SVM classifier	Class III	*Results* No threshold based on the value of a single feature can provide accurate glioma grading. The use of SBS to obtain the optimal feature set for the SVM classifier reduced classification error from 11.1 to 7.41% in differentiating Grade II vs III gliomas, and from 15.79 to 7.89% in differentiating Grade III and grade IV gliomas. The use of Random Forest selection further reduced the classification error to 3.7 and 5.26% in differentiating Grade II and III gliomas and grade III and IV gliomas, respectively *Author’s Conclusions* Classification error can be reduced when using an optimized feature set obtained from a combination of T1 perfusion parameters and volume of tumor components. Random forest selection provided better classification results compared to SBS when used for the SVM classifier. The combination of all features in an SVM provides lower classification error than each of the individual features, especially when redundant features are removed using a feature selection technique *Comments and Conclusions* The retrospective nature of the data acquisition provides class III data
Qu et al. [53]	*Study Description* Prospective, single institution *Patient Population* 72 histologically proven glioma patients (34 LGG, 38 HGG) from September 2015 to April 2018. All subjects underwent multi-b value DWI imaging and 3D pCASL prior to treatment *Treatment Regimen* The sensitivity and specificity of combined pCASL and stretched-exponential model in differentiating HGG and LGG was evaluated and compared to each technique alone. Two-tailed and Student t t-tests were used to compare the parameters between HGG and LGG. ROC curves were plotted for each parameter to determine which was best in glioma grading	Class III	*Results* The sensitivity and specificity of grading by conventional-enhanced MRI were 78.7 and 68.2%, respectively TBF, M-TBF, G-TBF, and W-TBF values derived from pCASL and stretched-exponential model were significantly higher in HGG than in LGG (*p* < 0.01). Parameters derived from these techniques showed higher SEN and SPE for grading than conventional gadolinium-enhanced MRI. SEN and SPE. SEN and SPE of combined g-TBF and α. Values were 94.1 and 98.7%, respectively. AUC values were 0.861 for TBF, 0.892 for M-TBF, 0.926 for G-TBF, 0.877 for W-TBF, 0.892 for α value, and 0.960 for combining G-TBF and α *Author’s Conclusions* 3D pCASL and stretched-exponential model can differentiate HGG and LGG. Combination of normalized G-TBF and α. Have higher sensitivity in glioma grading and can be used for noninvasive preoperative grading and follow-up *Comments and Conclusions* No meaningful contemporaneous or historical control cohort was provided. Therefore, this is class III data
Cao et al. [18]	*Study Description* Prospective, single institution *Patient Population* 57 pathologically confirmed glioma patients who underwent MRI. T1ρ and ADC coefficients were calculated *Treatment Regimen* The performance of T1ρ mapping in IDH characterization and tumor grading was evaluated and compared to that of ADC coefficients. Mann–Whitney U tests were conducted to compare ADC and T1ρ values between LGG and HGG, as well as between IDH1 mutants and wildtypes	Class III	*Results* ADC appears to be less strongly associated with tumor grade than T1ρ. T1ρ values of the solid and peritumoral edema areas were significantly higher in HGG compared with LGG (*p* < 0.001 and *p* = 0.005, respectively), ADC values did not show a significant difference between the two groups. Based on ROC analysis, T1ρ in the solid area were most effective at differentiating HGG and LGG (SEN: 80.6%, SPE: 81.0%). T1ρ value was also able to differentiate IDH wildtype and mutants (0.037) *Author’s Conclusions* T1ρ mapping has promise in assessing glioma grade and IDH status *Comments and Conclusions* No meaningful contemporaneous or historical control cohort was provided. Therefore, this is class III data
Yoon et al. [34]	*Study Description* Retrospective, single institution *Patient Population* 60 consecutive cerebral glioma patients (12 HGG and 48 LGG) between February 2003 and December 2006 *Treatment Regimen* All patients underwent multiparametric MR imaging and FDG-PET. The ability of multiparametric MR and FDG-PET to grade glioma was assessed in terms of their concordance rates. The PPV and NPV of concordant cases was evaluated. T tests or Mann–Whitney U tests was used to evaluate differences in multiparametric MR and FDG-PET results in their grading of cerebral gliomas	Class III	*Results* Parameters obtained from all imaging techniques were able to differentiate between HGG and LGG. rCBV ratio exhibited the greatest AUC (0.817) while the Cho/Cr ratio exhibited the lowest AUC value (0.694) in differentiating HGG and LGG. The double combinations of the five imaging techniques (MR DWI, MR PWI, H-MRS, FDG-PET, conventional MR) showed concordant results in 77.0% of cases, PPV in high-grade concordant cases was 97.3% while NPV in low-grade concordant cases was 70.2%. If at least two imaging parameters concordantly indicated HGG, the PPV was about 85% *Author’s Conclusions* Multiparametric MR and FDG-PET have concordant tendency in a two-tiered classification for the grading of cerebral gliomas and have potential in the assessment of cerebral glioma *Comments and Conclusions* The retrospective nature of the data acquisition provides class III data
Naveed et al. [14]	*Study Description* Retrospective, single center *Patient Population* 40 brain tumor patients with an oligodendroglial component. All patients had undergone conventional MRI and DWI, DSC, and MRS *Treatment Regimen* The diagnostic utility of combining various advanced imaging parameters in the differential diagnosis of oligodendrogliomas was studies. Parameter studied included ADC, rCBV, PS from DSC imaging, Cho/Cr ratio as well as Chol/NAA ratio from MRS. Mann–Whitney test was used to test mean values, ROC curves were generated for each parameter	Class III	*Results* Mean rPS was significantly different between grade II and II gliomas (0.7767 vs 3.7054, *p* = 0.007). While mean ADC values were lower in grade III than in grade II, this difference was not statistically significant (*p* = 0.121). When rPSmax obtained from the DSC maps was combined with the rCBVmax, there was a significant difference between grade II and III tumors (*p* < 0.03, AUC: 0.742). No significant differences between the Cho/Cr ratio or Cho/NAA ratio between tumor grades *Author’s Conclusions* Parameters derived from advanced imaging techniques have potential in tumor grading. PS values (rPSmax) were able to differentiate grades II and III gliomas. Combined rCBVmax and rPSmax can be used to noninvasively grade gliomas *Comments and Conclusions* The retrospective nature of the data acquisition provides class III data
Durmo et al. [32]	*Study Description* Retrospective, single center *Patient Population* 43 glioma patients (18 HGG, 10 LGG, 15 MET). Patients with meningioma, skull base lesions, and limited preoperative MR examinations were excluded *Treatment Regimen* All patients underwent pre and postoperative MRI. Volume and Diffusion metrics were calculated for each patient. Kruskal–Wallis tests were used for comparison by rank medians between the three groups as well as for pairwise comparisons between groups for biometrics. A binary logistic regression model was used, and ROC analysis and univariate and multivariate analyses were performed with SEN and SPE calculated for each biometric	Class III	*Results* KM analysis showed significant difference in overall survival between LGG (46.2 months), HGG (18.7 months, and MET (20.1 months) (*p* < 0.01). The biometric nCBF-T had the highest predictive capacity in differentiating HGG and LGG with a cutoff value of 4.35 AUC (SEN 93.3, SPE 100%, *p* < 0.001). Combining biometrics yielded an ROC curve with AUC = 1 (SEN 100%, SPE 100%, *p* < 0.001) in differentiating HGG an LGG *Author’s Conclusions* Normalized values of volumized, perfusion and diffusion biometrics were useful in differentiating LGG, HGG and MET. Combined biometrics had the most prominent cut off values in differentiating between the three groups, with nCBF-T being the single best biometric *Comments and Conclusions* The retrospective nature of the data acquisition provides class III data
Liu et al. [33]	*Study Description* Retrospective, single institution *Patient Population* 56 pathologically confirmed diffuse glioma patients with preoperative 3d pCASL and DWI between October 2015 and October 2017 *Treatment Regimen* The ability of 3dpCASL and DWI to differentiate tumor grades and predict IDH1 mutation status was analyzed. All patients had IDH1 mutation status assessed by pyrosequencing. Student’s t test and Mann–Whitney U tests were used to evaluate differences in DWI and 3D pCASL values between LGG and HGG as well as between mutant and wildtype IDH1 gliomas	Class III	*Results* CBFmax, CBF mean and rCBFmax values were all significantly higher in HGG (*p* < 0.05 for all) while ADCmean and ADCmin values were all significantly lower in the HGG. ADCmin with a cutoff value of 0.924 displayed the highest accuracy in distinguishing LGG and HGG with a SEN of 75.3% and SPE of 90.91%). CBFmean, max, rCBFmean and ADCmean all showed significant differences in mutant and wildtype IDH1 *Author’s Conclusions* Both 3dPCASL and DWI parameters were effective in distinguishing between LGG and HGG as well as differentiating IDH1 mutation status *Comments and Conclusions* The retrospective nature of the data acquisition provides class III data
Sakata et al. [31]	*Study Description* Single institution, retrospective *Patient Population* 49 newly diagnosed glioma patients who had undergone MR imaging with DWI and amide proton transfer imaging *Treatment Regimen* The value of amide proton transfer (APT) imaging in the preoperative grading of glioma was compared to that of 18F-FDG-PET and DWI. ROC curve analysis was used to assess the ability of imaging parameters in differentiating HGG and LGG. The added value of APT imaging in addition to FDG-PET and DWI was assessed using NRI analyses	Class III	*Results* No significant differences in the parameters derived from each imaging method in differentiating HGG and LGG. When APT mean was combined with T/N ratio, the NRI was 0.64 (*p* = 0.04) for diagnosis of HGG and 0.95 (0.001) for the diagnosis of glioblastoma. When ADCmin was combined with T/N, the NRI was 0.43 (*p* = 0.16) for diagnosis of HGG and 1.36 (*p* < 0.001) for the diagnosis of glioblastoma *Author’s Conclusions* APT showed the potential to improve diagnostic performance in the identification of HGG. APTmean offers good diagnostic accuracy for HGG, comparable to that of other single imaging biomarkers such as ADCmin or T/N ratio from 18-FDG PET. Multiparametric analysis including APT, and FDG-PET can improve the classification of glioma *Comments and Conclusions* The retrospective nature of the data acquisition provides class III data
Saini et al. [55]	*Study Description* Retrospective, multi-center *Patient Population* 129 treatment-naïve pathologically confirmed glioma patients. All patients underwent MRI, SWI and T1-perfusion MRI *Treatment Regimen* The use of rCBV values derived from T1 PMRI and SWI derived from ITTS in differentiating grades of glioma was studied. Kruskal–Wallis ANOVA was used to measure the differences of rCBV values between the three tumor grades, Mann–Whitney U tests was used for pairwise comparison of groups. Fisher’s exact test was used for comparison of ITSS scores	Class III	*Results* Significant differences were found in rCBV values among the three different glioma grades (*p* < 0.001). rCBV values were able to differentiate grade II and III gliomas (*p* < 0.001) but was less accurate in differentiating grade III and IV gliomas. ITSS scores were able to differentiate grade III and IV gliomas (*p* < 0.001), but not between grade II and III gliomas. ROC analysis showed the highest AUC (0.85) for the combined rCBV and ITSS in differentiating grade II + III from grade IV gliomas *Author’s Conclusions* Combining T1 perfusion and SWI parameters can be useful in differentiating glioma grade. T1-perfusion derived rCBV can effectively differentiate grade II and grade III gliomas but is less effective in differentiating grade III and IV gliomas. Combining DWI derives ITSS with rCBV has potential in differentiating grade III and IV gliomas *Comments and Conclusions* The retrospective nature of the data acquisition provides class III data
Stadlbauer et al. [60]	*Study Description* Retrospective, single institution *Patient Population* 83 patients with pathologically confirmed glioma *Treatment Regimen* Biomarker maps were generated for the oxygen extraction fraction and cerebral metabolic rate of oxygen. The diagnostic performance of oxygen metabolism and neovascularization activity for grading and characterization of IDH gene mutation in gliomas was studied. Linear regression was used for correlations between biomarkers and WHO grades. Analysis of variance was used to elucidate differences between imaging biomarkers in WHO grades and IDH gene mutation	Class III	*Results* Only MTI was significantly different between IDH mutation and wild type (*p* = 0.013). LGG showed areas of increased OEF (+ 18%, *p* < 0.001) while grade III and IV showed decreased OEF when compared with normal brain tissue (− 54%, *p* < 0.0021) and − 49%, *p* < 0.001), respectively *Author’s Conclusions* Biomarkers such as oxygen metabolism and MTI are useful in differentiating glioma grade as well as IDH mutant and wildtype. This is in line with the accepted knowledge of glioma-associated neovascularization, where LGG grow along preexisting vessels while HGG start to generate their own tumor vessels *Comments and Conclusions* The retrospective nature of the data acquisition provides class III data
Leu et al. [38]	*Study Description* Retrospective, single center *Patient Population* 65 histologically confirmed grade II or III glioma patients. All patients had undergone DSC perfusion-weighted MRI, diffusion-weighted MRI, T2-weighted and post contrast T1-weighted anatomical scan performed at initial diagnosis prior to surgery. All patients had known IDH1 and 1p/19q co-deletion status *Treatment Regimen* The value of parameters derived from perfusion and diffusion-weighted MRI in differentiating tumors based on the 2007 WHO glioma classification scheme (astrocytoma vs oligodendroglioma) and genetic subtypes according to the 2016 WHO reclassification was compared. Bootstrap hypothesis testing was used for all tests due to the small sample size. Logistic regression was used to classify IDH and 1p19q- status using median rCBV, median ADC, presence or absence of contrast enhancement and volume of T2-enhancment using an in-house MATLAB code	Class III	*Results* Neither rCBV nor ADC differed significantly between pure astrocytomas and pure oligodendrogliomas. IDH wild types had a significantly higher rCBV (1.21 vs 0.6) and significantly lower ADC (0.9 vs 1.7) compared to mutants. ADC alone was significantly different between IDH mutants and wildtypes (*p* = 0.0030), the presence or absence of contrast enhancement trended towards significance (*p* = 0.0060). The combined multivariate model including all four parameters improved the ability to differentiate wildtype and mutant with high SEN and SPE (74, 79%, *p* < 0.0001). ADC was significantly difference between 1p19q wildtype and mutant (*p* = 0.0018) *Author’s Conclusions* ADC better differentiated between genetic subtypes of glioma according to the 2016 WHO guidelines compared to the classification scheme based on histological features outlined in the 2007 WHO guidelines. ADC, especially in combination with rCBV, T2 volume enhancement and contrast enhancement allows for the differentiation of both IDH and 1p19q wildtypes and mutants *Comments and Conclusions* The retrospective nature of the data acquisition provides class III data
Lin et al. [30]	*Study Description* Retrospective, single institution *Patient Population* 33 histologically confirmed oligodendrogliomal tumor patients with IDH-mutated and ap/19q co-deleted ODs. All patients underwent MRI scans combined with DWI, SWI, and DSC-PWI *Treatment Regimen* The ability of parameters such as nADC, ITSS, nCBV, and nCBF derived from DWI, SWI, and DSC-PWI to differentiate between low- and high-grade oligodendrogliomas in this set of patients was studied. Parameters between low-and high-grade ODs were compared using Mann–Whitney U tests. SEN, SPE, PPV and NPV for each parameter was calculated based on the optimal threshold for each parameter	Class III	*Results* IDH mutant and 1p/19q co-deleted ODs can be graded using the parameters derived from cMRI and aMRI techniques. nADC, ITSSs, nCBV and nCBF were all significantly different between low-and high-grade ODs. The combination of SWI and DSC-PWI resulted in the highest SEN and SPE (100 and 93.33%, respectively) *Author’s Conclusions* MRI techniques such as cMRI and aMRI can effectively stratify IDH-mutant and 1p/19 co-deleted ODs by grade *Comments and Conclusions* The retrospective nature of the data acquisition provides class III data
Neill et al. [39]	*Study Description* Prospective, single institution *Patient Population* 122 surgically treated recurrent grade II glioma patients *Treatment Regimen* Image-guided tissue samples were obtained during resection. The use of parameters derived from MR anatomic, diffusion and spectroscopic in predicting prognosis was evaluated. KM curves were plotted to test for differences in PFS between grades at recurrence	Class III	*Results* Gliomas that recurred as grade IV had significantly shorter PFS than those that recurred as grade II (*p* < 0.05). Parameters derived from MR anatomic, diffusion and spectroscopic were the volumes of T2. All and T1. ROIS were significantly associated with PFS. All MR parameters except 10% nADC and sum (nLAC) were significantly predictive of PFS in astrocytomas while only nFA parameters were predictive in oligodendroglioma. nADC values were negatively correlated with malignant tissue characteristics *Author’s Conclusions* Parameters derived from multi-parametric provide a non-invasive means of assessing prognosis in patients with recurrent low-grade gliomas *Comments and Conclusions* No meaningful contemporaneous or historical control cohort was provided. Therefore, this is class III data
Togao et al. [24]	*Study Description* Prospective, single center *Patient Population* 45 consecutive patients with diffuse glioma (16 LGG and 29 HGG) who underwent a subsequent resection or biopsy *Treatment Regimen* Patients underwent IVIM imaging as well as DSC PW MRI. rCBV mapswere generated and normalized to contralateral normal-appearing white matter to yield rCBV maps. The relationship between the parameters f and rCBV was measured using Pearson correlation. The diagnostic accuracy of the parameters D, ADC, D* and f in differentiating LGGs from HGGs was evaluated using AUC curves	Class III	*Results* Interobserver agreement for D and f-values showed excellent agreement with an ICC of 0.90 and r of 0.79 (*p* < 0.0001) for ADC, an ICC of 0.95 and r of 0.92 (*p* < 0.0001) for D*, and an ICC of 0.94 and r of 0.91 (*p* < 0.0001) for the f-values. D values of grade III (*p* < 0.05) and grade IV (*p* < 0.01) gliomas were significantly lower than those of grade II gliomas, while D* did not differ among the different grades. F values of grade III (*p* < 0.001) and grade IV (*p* < 0.001) were significantly higher than those of grade II gliomas. rCBV values of grade III (*p* < 0.01) and grade IV (*p* < 0.001) gliomas were significantly higher than those of grade II gliomas. F values showed highest diagnostic performance in discriminating with AUC values of 0.95, while D* showed a low diagnostic performance *Author’s Conclusions* Diffusion and Perfusion parameters of IVIM imaging are useful in differentiating HGGs form LGGs. F values were most useful in differentiating HGG and LGG, while D* was the least useful *Comments and Conclusions* No meaningful contemporaneous or historical control cohort was provided. Therefore, this is class III data
Arevalo-Perez et al. [29]	*Study Description* Retrospective, single-institution analysis of patients with baseline DWI and DCE-MRI *Patient Population* 63 patients with pathologically confirmed low-or high-grade glioma *Treatment regimen* All patients had baseline DWI and DCE-MRI. Volumetric analysis was conducted to assess the diagnostic accuracy of parameters derived from these Mann–Whitney U test was to compare differences between each when comparing high- and low-grade gliomas in terms of Vp, Ktrans, and mean and minimum ADC values	Class III	*Results* Vp, Ktrans values from both DCE-MRI and ADC from DWMRI were useful in differentiating between low and high grade as well grade I, II, and II gliomas. Vpmean was the best at differentiating HGG and LGG (AUC 0.974, SEN 90.7%, SPE: 95%) *Author Conclusions* Histogram analysis of T-1 weighted perfusion parameters Vp and Ktrans from DCE-MRI and ADC from dMRI can assess glioma grade., with Vpmean being the best predictor *Comments and Conclusions* The retrospective nature of the data acquisition provides class III data
Lotumolo et al. [40]	*Study Description* Retrospective, single center *Patient Population* 80 glioma patients (48 HGG and 32 LGG) who received DWI sequences and MRS before and after surgery *Treatment Regimen* 80 patients were assessed retrospectively. The ability of ADC coefficients and metabolites ratios derived from MRS and DWI to assess the progression and regression of tumors was compared. Student-t test was used to evaluate changes in ADC values. Pearson’s correlation was used to examine statistical correlations between these parameters before and after therapy	Class III	*Results* In LGG, MRS showed a SEN, SPE, PPV, and NPV of 33.3, 80, 50, and 66.6%, respectively in predicting reduction of disease after therapy based on the Cho/NAA ratio. The corresponding values for DWI were 83.3, 90, 83.3, and 90% based on the Cho/NAA ratio. In HGG, MRS showed a greater accuracy (83.3 vs 75%) in predicting reduction of disease after therapy *Author’s Conclusions* MRS appears to more accurately predict response to treatment in HGG while DWI appears to better predict response to treatment in LGG *Comments and Conclusions* The retrospective nature of the data acquisition provides class III data
Li et al. [50]	*Study Description* Retrospective, single institution *Patient Population* 32 patients (15 with LGG, 17 with HGG) who underwent conventional MRI, DCE-MRI, and SWI before surgical resection *Treatment Regimen* The ability of Ktrans values, Ve, and degree of ITSS derived from DCE-MRI and non-contrast enhanced SWI to differentiate between HGG and LGG was evaluated. Spearman’s correlation analysis was used to determine associations between these parameters	Class III	*Results* Ktrans, Ve, and ITSS helped distinguish between HGG and LGG. Ktrans (0.117 vs 0.026) and Ve (0.505 vs 0.121) values were significantly higher in HGGs than in LGGs (*p* < 0.001). Degree of ITSS of LGGs was significantly lower than that of HGGs (1.2 vs 2.6, *p* < 0.01). Ktrans values were strongly correlated with Ve values (r = 0.823, *p* < 0.01) and moderately correlated with degree of ITS (4 = 0.473, *p* < 0.01). A cut-off value of 0.054 for Ktrans provided the best combination of SEN (04.1%) and SPE (93.3% in differentiating HGG and LGG *Author’s Conclusions* Ktrans, Ve, and ITSS derived from DCE-MRI were effectively able to differentiate between grades of glioma. A moderate correlation between Ktrans and ITSS in the same glioma segment was found *Comments and Conclusions* The retrospective nature of the data acquisition provides class III data
Fudaba et al. [35]	*Study Description* Retrospective, single institution *Patient Population* 32 histologically confirmed patients with grade II and III glioma between March 2010 and October 2012 *Treatment Regimen* 32 patients were examined retrospectively. A variety of parameters derived from PASL, DTI, and MR spectroscopy such as relative cerebral blood flow, fractional anisotropy and the Cho/Cr, NAA/Cho, NAA/Cr, and lactate/Cr ratios were evaluated to determine whether these variables were useful in grading between cerebral gliomas. The correlations between these parameters and the proliferation marker, Ki-67, was also evaluated. The turkey-Kramer test was used to compare these parameters in each of the three groups	Class III	*Results* There was a significant negative correlation between the minimum ADC ratio and the Ki-67 index (r = − 0.470, *p* = 0.0089). There was a significant positive correlation between the Cho/Cr ratio and the Ki-67 index (r = 0.461, *p* = 0.0103) and between the Lac/Cr ratio and the Ki-67 index (*p* = 0.0199). The combination of minimum ADC ratio and Cho/Cr ratio exhibited the greatest diagnostic accuracy in differentiation between HGG and LGG (SE = 87.0, 88.9%) *Author’s Conclusions* PASL, DTI, and MR spectroscopy are useful parameters in predicting the malignancy of cerebral gliomas. The parameters derived from these also correlated with the proliferative potential of gliomas *Comments and Conclusions* The retrospective nature of the data acquisition provides class III data
Caulo et al. [27]	*Study Description* Retrospective, single institution *Patient Population* 118 pathologically confirmed glioma patients that underwent MR imaging from January 2008 to September 2012 *Treatment Regimen* Each patient underwent a semiquantative analysis based on the report at initial presentation, a quantitative analysis performed in consensus by two different radiologists based on MR imaging sequences, and a quantative analysis in five different tumor regions. The ability of parameters derived from this such as Cho/Cr, NAA/Cr, Cho/NAA, lactate/CR and lipids/Cr to differentiate between glioma grades was evaluated. T tests were used to evaluate differences in each parameter between HGG and LGG. Discriminant function analysis was used to determine which variables were effective in predicting glioma grade	Class III	*Results* rCBV in contrast-enhanced regions (SE: 80%, SPE 91%), areas of low signal intensity on T2 (SEN: 57%, SPE: 97%), restricted diffusivity regions (SE: 54%, SPE: 97%) and choline/creatine ratio in regions with the lowest signal intensity (SE: 49%, SPE: 88%) were collectively able to correctly grade 95% of patients *Author’s Conclusions* Parameters from multiparametric MR imaging can effectively discriminate HGG and LGG *Comments and Conclusions* The retrospective nature of the data acquisition provides class III data
Wang et al. [54]	*Study Description* Retrospective, single institution *Patient Population* 94 patients with histologically proven astrocytoma who underwent SWI, DSC, and conventional MR before biopsy *Treatment Regimen* The use of susceptibility weighted imaging (SWI) without contrast to grade astrocytomas was measured. ITTS and rCBV max derived from SWI was compared to DSC PWI in the grading of brain astrocytomas. Kruskal Wallis tests used to compare mean ITSS degrees, Welch test was used to compare differences in the rCBV max. Spearman correlation coefficients were used to assess correlation between these parameters and tumor grade	Class III	*Results* ITSS was significantly different between the three grades of astrocytoma (*p* < 0.01) based on the Kruskal Wallis test. rCBV max were significantly different among the three astrocytoma grades based on the Welch test (*p* < 0.01). ITSS showed significant correlation with rCBV max within astrocytomas (r = 0.72, *p* < 0.01). AUC for ITSS and rCBV max for differentiating high-and low-grade astrocytomas was comparable (0.999 vs 0.992) *Author’s Conclusions* Nonenhanced SWI and MR perfusion-weighted imaging are comparable for astrocytoma grading. This indicates that conventional MR and SWI can be used for astrocytoma grading in patients who cannot tolerate contrast agents *Comments and Conclusions* The retrospective nature of the data acquisition provides class III data

*MRI* magnetic resonance imaging, *SVM* support vector machine, *SBS* sequential backward selection, *DWI* diffusion-weighted imaging, p*CASL* pseudo-continuous ASL, *HGG* high-grade glioma, *LGG* low-grade glioma, *ROC* receiver operating characteristics, *M-TBF* contralateral mirror regions, *G-TBF* normal grey matter, *W-TBF* white matter, *SEN* sensitivity, *SPE* specificity, *α* diffusion heterogeneity, *T1ρ* spin–lattice relaxation in the rotation frame at the presence of an external RF pulse in the transverse plane, *ADC* apparent diffusion coefficient, *IDH* isocitrate dehydrogenase, *AUC* area under the curve, *FDG* fluorodeoxyglucose, *PET* positron emission tomography, *Cho* choline, Cr creatinine, *PPV* positive predictive value, *NPV* negative predictive value, *Ktrans* volume transfer constant, *Vp* plasma volume, *DSC* dynamic susceptibility contrast, *PW* perfusion weighted, *CBV* cerebral blood volume, *pCASL* pseudo-continuous arterial spin labeling, *MRS* magnetic resonance spectroscopy, *rPs* relative permeability surface product, *NAA* N-acetylaspartate, T/N tumor to normal ratio, *NRI* net reclassification index (NRI), an index that shows how well a new model reclassifies subjects, *SWI* susceptibility weighted imaging, *PMRI* perfusion MRI, *ITSS* intratumoral susceptibility signals, *MTI* microvessel type indicator, Ve Interstitial volume fraction, PS permeability surface area product, *MET* metastasis, KM Kaplan Meier, *nCBF* normalized cerebral blood flow, *WHO* World Health Organization, *OEF* oxygen extraction fraction, *OD* oligodendroglioma, *nCBV* normalized maxim CBV, *CBF* cerebral blood flow, *nCBFmax* normalized maximum CBF, *cMRI* conventional MRI, *aMRI* advanced magnetic resonance imaging, *nADC* normalized ADC, *PFS* progression-free survival, *ROI* region of interest, *nLAC* levels of Lactate normalized by the median level of NAA from voxels within the selected volume, *nFA* normalized fractional anisotropy, *IVIM* intravoxel incoherent motion, R correlation coefficient, *ICC* intraclass correlation coeffecient, D* fast diffusion coefficient, *D* slow diffusion coefficient, *f* fraction of fast ADC, *Ki-67* mitotic index, *PASL* pulsed arterial spin labeling, *DTI* diffusion tensor imaging, *DCE* dynamic contrast enhanced

**Table 7:** Amino Acid PET: diagnosis, prognosis, monitoring

Author and year	Description	Data Class	Conclusions
Nomura et al. (2018) [74]	*Study Description* Retrospective, single center *Patient Population* 168 newly diagnosed brain who underwent MET-PET between March 2012 and October 2015 *Treatment Description* The role of MET-TAC obtained from MET-PET dynamic study with 3D-PET in the differential diagnosis of brain tumors was studied. The role of this modality in aiding in the understanding of tumor biological activity and vascular in each common brain tumor was also evaluated. ANOVA testing was used to compare MET-SUV among tumors	Class III	*Results* MET-SUVs were significantly higher in early and late phases in GBM compared to anaplastic astrocytoma, diffuse astrocytoma, and the normal frontal cortex. No significant difference between anaplastic astrocytoma and diffuse astrocytoma in MET-SUV. MET SUV was higher in the initial phase and had tendency to increase more in the early phases of anaplastic oligodendroglioma compared to oligoastrocytoma, anaplastic oligoastrocytoma, and oligodendroglioma. Time to peak was significantly shorter in low grade tumors that included an oligodendroglial component (oligodendroglioma or oligoastrocytoma) compared to diffuse astrocytoma (*p* < 0.05). A cutoff of the TTP to 15.5 min for the differentiation of tumors that included an oligodendroglial component from astrocytic tumors had a SEN of 77.4% and a SPE of 54.4% *Author’s Conclusions* Quantification of the MET-TAC obtained from dynamic MET-PET study could be helpful in the non-invasive discrimination of brain tumor subtypes *Comments and Conclusions* The retrospective nature of the data acquisition provides class III data
Bashir et al. (2018) [71]	*Study Description* Retrospective, single institution *Patient Population* 42 patients with pathologically confirmed LGG that had previously been treated who presented with signs suggestive of tumor progression *Treatment Regimen* All patients underwent neurosurgical intervention following FET-PET assessment. The accuracy of FET PET in detecting malignant progression was evaluated using ROC curve analysis. Predictors influencing the accuracy of FET PET were assessed multiple linear regression. Criteria for tumor progression was based on the RANO criteria	Class III	*Results* ROC analysis of the imaging PET or contrast-enhanced MRI did not significantly differentiate malignant transformation of LGG. Interindividual variability in FET uptake in different WHO groups was high, and a reliable cut off value could not be determined for TBRmax and mean using ROC analysis. Removal of certain confounding factors (oligodendroglial group, previous oncological treatment, and combination of FET PET parameters) FET PET demonstrated significantly increased ability to detect malignant transformation of LGGs (SEN: 75%, SPE: 83%, AUC: 0.828, *p* = 0.003) *Author’s Conclusions* FET PET alone is not adequate to replace histological confirmation in assessing transformation of LGGs *Comments and Conclusions* The retrospective nature of the data acquisition provides class III data
Kim et al. (2017) [79]	*Study Description* Prospective, single center *Patient Population* Seventy-three patients with surgically confirmed cerebral gliomas (19 grade II, 21 grade III, and 33 grade IV) underwent PET/CT prior to surgery *Treatment Regimen* All patients underwent 11C-acetate PET/CT prior to surgery. The role of 11C-acetate PET/CT in predicting histologic grades, progression-free survival (PFS) and overall survival (OS) was evaluated. Kruskal–Wallis test was used to assess the relationship between tumor-to-choroid plexus ratio (TCR) derived from PET/CT and tumor grade. A Cox PH model was used to assess the relationship of these parameters on survival	Class III	*Results* Median TCR was 1.20 (interquartile range [IQR], 1.14 to 1.4) in grade II, 1.65 (IQR, 1.26 to 1.79) in grade III, and 2.53 (IQR, 1.93 to 3.30) in grade IV gliomas. Significant differences in TCR were seen between the three WHO grade groups (*p* < 0.001). TCR was prognostic for PFS (*p* = 0.016) and for OS (*p* = 0.024). TCR was an independent prognostic factor for PFS. There were significant differences in TCR among the three tumor grades (*p* < 0.001). Median OS in patients with a TCR > 1.6 was 25 months, while it was 64 months in patients with a TCR < 1.61 (*p* < 0.0001). TCR was significant predictor for PFS (*p* = 0.0016). Median PFS was 22 months in TCR > 1.61 and 57 months in TCR < 1.61 (*p* < 0.001) *Author’s Conclusions* Higher 11C-acetate uptake is an independent prognostic factor for PFS. TCR was better at predicting survival than WHO grade *Comments and Conclusions* No meaningful contemporaneous or historical control cohort was provided. Therefore, this is class III data
Bund et al. (2017) [66]	*Study Description* Prospective, single institution *Patient Population* 53 glioma patients (35 LGG and 18 HGG) *Treatment Regimen* Each patient underwent static FDOPA PET at 30 min and had MRSI with measurements of various metabolites ratio to evaluate the value of this modality in assessing primary brain tumor aggressiveness	Class III	*Results* FDOPA was effective in discriminating dysembryoplastic neuroepithelial tumor and grade II oligodendroglioma (*p* < 0.01). An SUV_max_ (T/N)_30_ of 2.16 achieved a SEN, SPE, PPV, and NPV of 60,100,100, and 83.33%, respectively in differentiating between low and high-grade gliomas *Author’s Conclusions* Data from amino acid metabolism alone or in conjunction with MRSI allows for effective discrimination between dysembryoplastic neuroepithelial tumor and grade oligodendroglioma as well as between HGG and LGG without contrast enhancement on MRI *Comments and Conclusions* The lack of randomization or identification of a contemporaneous cohort of patients imaged without PET leaves this as class III data
Rossi-Espagnet et al. (2016) [41]	*Study Description* Retrospective, single center *Patient Population* 12 pathologically proven LGG patients between January 2012 and December 2015 *Treatment Regimen* All Patients underwent DMRI, PMRI and 18F-FDOPA-PET. The role of 18F-FDOPA PET in estimating progression at follow up was evaluated and compared to that of advanced MR sequences such as DSC and DWI. Pearson’s correlation test was used to test the relationship between PET and MR measurements. ROC analysis was performed to assess SEN and SPE for parameters reaching statistical significance. A multivariate analysis of variance was used to further test the predictive ability of each parameter	Class III	*Results* No significant correlation between PET parameters and ADC or relative CBV values. There was a significant correlation between follow-up status (stable vs progressed) and T/Nmax (*p* < 0.05) derived from PET. At a cut-off value of 1.7, this had a SEN of 83% and SPE of 100% in assessing progression *Author’s Conclusions* 18F-FDOPA-PET showed a significant prognostic role in the follow-up of LGGs. T/Nmax ratio was the best parameter at predicting prognosis. MRI. MRI with perfusion and diffusion techniques do not correlate with 18F-FDOPA PET and provide different information *Comments and Conclusions* The retrospective nature of the data acquisition provides class III data
Bette et al. (2016) [67]	*Study Description* Retrospective, single center *Patient Population* 65 histologically confirmed LGG patients between November 2006 and March 2015 who underwent MRI and FET-PET before resection *Treatment Regimen* Correlations between ^18^F-FET-PET derived volumes and patterns with MR characteristics on ^18^F-FET-PET with histological parameters and progression-free survival were assessed. Differences between the two groups were assessed using the Mann–Whitney U test, correlations were calculated by the χ^2^ test	Class III	*Results* 78.5% of LGGs showed elevated tracer uptake in ^18^F-FET-PET. High sensitivity of ^18^F-FET-PET trace uptake was found for 1p19q codeletion, IDH1/mutation, elevated Ki67, contrast enhancement, and p53 mutation was found for low TBRs. Sensitivity declined with greater TBR. Specificity was low for all parameters but increased with greater TBR. SEN, SPE, PPV and NPV for IDH1/2 mutation at a TBR > 1.6 was 71.4, 52.6, 73.5, and 50.0%, respectively *Author’s Conclusions* FET-PET provides diagnostic information on LGG, as 78.5% of them showed PET trace uptake. No further significant correlation between trace uptake, histologic features, survival, or IDH1/2 mutation status *Comments and Conclusions* The retrospective nature of the data acquisition provides class III data
Villani et al. (2015) [78]	*Study Description* Prospective, single center *Patient Population* 50 histologically confirmed newly diagnosed glioma patients *Treatment Regimen* All patients underwent PET (18F)-FDOPA and MRI at baseline and every 6 months thereafter. The ability of PET (18F)-FDOPA to predict eventual radiological progression in this population was evaluated. A Cox PH model was used to examine the value of PET (18F)-FDOPA in predicting radiological progression. ROC curves were constructed to determine the optimal maximum standardized uptake value ratio cut-off value	Class III	*Results* A maximum standardized uptake value greater than 1.75 was associated with a HR of 9.1905 (*p* = 0.005) for disease progression. The best predictive cut-off value for maximum standardized uptake value in predicting disease progression was 1.75, with a SEN, SPE, PPV, and NPV of 0.769, 0.824, 0.625, and 0.914, respectively. Disease duration (HR 0.66 for each year, *p* = 0.025) was also an independent predictor of disease progression *Author’s Conclusions* PET (18F)-FDOPA may play a role in the evaluation of LGG. respectively *Comments and Conclusions* No meaningful contemporaneous or historical control cohort was provided. Therefore, this is class III data
Tietze et al. (2015) [28]	*Study Description* Retrospective, single institution *Patient Population* 13 patients (7HGG and 6LGG) *Treatment Regimen* The diagnostic value of spatial tumor distribution derived from pMRI and dMRI was evaluated and compared to that of MET-PET. The accuracy of all three methods were compared by calculating AUCS	Class III	*Results* CBV maps derived from perfusion data were significantly more accurate than cMRI in predicting high MET uptake (AUC 0.76 vs 0.657. While CBV maps were comparable to MET-PET in 5/7 cases of HGG, they were insufficient in all cases of LGG. ADC maps and cerebral blood flow maps did not further improve in accuracy *Author’s Conclusions* pMRI can increase the diagnostic accuracy of cMRI when added to the presurgical protocol in HGG. Defining LGG with subtle or no alterations on cerebral blood volume maps remains a challenge *Comments and Conclusions* The retrospective nature of the data acquisition provides class III data
Janvier et al. (2015) [77]	*Study Description* Retrospective, single center *Patient Population* 31 patients with histological, radiological, or clinical proof of DLGG *Treatment Regimen* All patients had histological diagnosis of grade II, III, or IV glioma obtained less than 1 year from the PET study. Spearman correlation coefficients were used to determine if there was a correlation between e F-DOPA uptake and tumor grade. The Mann–Whitney U test was used to determine the best SUV-derived indices	Class III	*Results* There was no statistically significant difference between the 2 groups in terms of mean tumor volume between HGGs and LGGs (77 vs 41 cm^3^, *p* > 0.05). All SUV indices except for SUVmax of isocontoured volume at 50% of the peak voxel intensity. allowed for discrimination of LGG and HGG. The best correlated indices were SUVmean T/N and SUVmean T/S, with spearman correlation coefficients of 0.561, and 0.522, respectively *Author’s Conclusions* F-FDOPA PET is easily able to discriminate LGG from HGG with just one acquisition *Comments and Conclusions* The retrospective nature of the data acquisition provides class III data
Jeong et al. (2015) [36]	*Study Description* Prospective, single center *Patient Population* 10 pathologically verified glioma patients (5 WHO grade II, 5 WHO grade IV) *Treatment Regimen* MRI and AMT-PET images were acquired for all patients. The role of isotropic diffusion spectrum imaging (IDSI) with independent component analysis (ICA) in assessing the cellularity and grade of tumor was assessed. ROC curves were generated to assess accuracy DWI and IDSI derived cellularity in tumor grading. Pearson’s correlation test was used to assess the relationship between Ki-67 and DWI-ADC and IDSI derived cellularity	Class III	*Results* IDSI-derived cellularity was elevated in both FLAIR and AMT-PET-derived regions of HGG. IDSI-derived cellularity showed a slightly higher probability to differentiate HGG from LGG compared with the DWI-ADC. ROC curve found that IDSI-derived cellularity was effective in differentiating HGG and LGG (SEN: 80%, SPE: 80%). ADC and IDSI-derived cellularity were significantly correlated with glioma proliferative index (*p* < 0.001) *Author’s Conclusions* IDSI demonstrates potential as a measure for DTI studies to assess hypercellularity in malignant gliomas. IDSI-MRI along with AMT-PET aid in the pretreatment assessment of glioma *Comments and Conclusions* No meaningful contemporaneous or historical control or validation cohort was provided. Therefore, this is class III data
Thon et al. (2015) [70]	*Study Description* Prospective, single institution *Patient Population* 98 consecutive patients with MRI-suspected LGG. All patients exhibited increased 18F-FET uptake as compared to the corresponding area in the nonaffected contralateral hemisphere *Treatment Regimen* TAC patterns derived from 18-F-FET-PET in terms of PFS was examined. KM curves and PH models were conducted to measure the effects of parameters on PFS. The ability of TAC parameters to differentiate glioma grade was measured using Kruskal–Wallis test and Wilcoxon test	Class III	*Results* The three distinct TAC patterns identified were homogeneous increasing, focal decreasing, and homogenous decreasing. Patients with homogenous increasing TAC were less likely to suffer from tumor progression compared to patients with focal decreasing TAC (*p* = 0.007). PFS at 1 year for homogenous increasing, focal decreasing, and homogenous decreasing TAC were 92, 89, and 50%, respectively (*p* = 0.002). HR for patients with increasing TAC for disease progression was 0.22 *Author’s Conclusions* TAC patterns derived from Dynamic 18-F-FET-PET imaging is indicative of patient prognosis. Each of the three TAC patterns was associated with a different prognosis. TAC analysis may be useful as an independent imaging biomarker *Comments and Conclusions* No meaningful contemporaneous or historical control cohort was provided. Therefore, this is class III data
Gempt et al. (2015) [68]	*Study Description* Retrospective, single study *Patient Population* 152 patients who underwent resection or biopsy of newly diagnosed intracranial glioma and had preoperative FET PET and standard MRI *Treatment Regimen* The ability of the tumor-to-normal (T/N) ratio derived from FET-PET to grade and estimate the prognosis of gliomas was evaluated. Mann–Whitney U tests were used to compare LGG and HGG in terms of T/N ratio. ROC curves were calculated to evaluate accuracy of T/N in differentiating LGG and HGG. KM curves were used to estimate overall survival	Class III	*Results* Median T/N ratio in LGG was 1.65, while it was 3.14 in HGG (*p* < 0.001). No deaths were recorded for a T/N ratio < 1.6, while median survival for T/*N* > 3 was 14.0 months (*p* < 0.001). ROC curve for differentiation of HGG and LGG showed an AUC of 0.903. Optimal cut-off to differentiate HGG and LGG was a T/N ratio of 2.26 (SEN 0.79, SPE 0.88) *Author’s Conclusions* T/N ratio derived from FET-PET is useful in the grading and prognostication of glioma *Comments and Conclusions* The retrospective nature of the data acquisition yields class III data
Jansen et al. (2014) [69]	*Study Description* Retrospective, single center *Patient Population* 59 newly diagnosed supratentorial WHO grade II astrocytoma who had undergone FET-PET *Treatment Regimen* The correlation between FET-PET uptake over time (increasing vs. decreasing time-activity curves) and overall survival (OS), progression-free survival (PFS), and time to malignant transformation (TTM) was investigated. Influence of categorical variables on these outcomes was measured using the log-rank test of the KM curve	Class III	*Results* Rate of OS, PFS, and TTM did not differ between FET positive and FET-negative gliomas. Chemo or radiotherapy was applied more often to FET-positive glioma (*p* = 0.003). In patients with FET-positive gliomas, increasing time to peak on time-activity curves resulted with significantly longer PFS (*p* < 0.01), TTM (*p* < 0.001), and OS (*p* = 0.002) *Author’s Conclusions* Dynamic acquisition of FET-PET scans enables the identification of high risk LGG and can be implemented for optimized treatment management. FET-negative LGG do not typically have a benign course *Comments and Conclusions* The retrospective nature of the data acquisition provides class III data
Santoni et al. (2014) [75]	*Study Description* Retrospective, single center *Patient Population* 53 primary glioma patients with Karnofsky Performance Status > 70. All patients underwent MET-PET between January 2006 and June 2011 *Treatment Regimen* The SEN and SPE of MET-PET in detecting malignant progression from low grade to anaplastic astrocytoma was evaluated. The role of this modality in assessing response to temozolomide therapy for anaplastic astrocytoma and glioblastoma patients was also studied. Comparisons of uptake between histological groups was performed using the Kruskal–Wallis nonparametric test	Class III	*Results* MET-PET was able to detect progression of low trade to anaplastic astrocytoma with a sensitivity of 91.56% and a specificity of 95.18%. In patients with a histological diagnosis of GBM treated with surgery and concomitant radiochemotherapy and adjuvant TMZ, 11C-MET PET had a sensitivity of 96.52% in tumor assessment during TMZ therapy with no false positives. Mean uptake in low-grade astrocytomas, anaplastic astrocytomas and GBMs was 1.73, 1.99, and 2.24, respectively (p-0.0009) *Author’s Conclusions* MET-PET enabled the assessment of post-surgery status in both low and anaplastic astrocytomas and early detection of malignant progression in low grade astrocytoma patients with high sensitivity and specificity. It was also effective in the early detection of recurrence of anaplastic astrocytoma and GBM patients treated with TMZ. The potential impact of these findings should be further investigated in randomized trials *Comments and Conclusions* The retrospective nature of the data acquisition provides class III data
Belohlavek et al. (2014) [72]	*Study Description* Retrospective, single institution *Patient Population* 41 histologically confirmed untreated LGG patients between August 2009 and September 2012 *Treatment Regimen* All patients underwent FLT-PET. The parameters SUVmax, SUVmean, SUVpeak TBR, TBRmax, and TBR were calculated. The ability of these parameters to predict overall and event-free survival was examined using stepwise COX PH regression. ROC curve analysis was used to determine the optimal parameter in predicting overall survival	Class III	*Results* Increased FLT uptake was strongly correlated with survival. With a cut-off value of 0.236 for SUVmean, the hazard ratio for overall survival of the test positive group over the test negative group was 17.6 All parameters exhibited significant ability to predict whether patients survive or die during the study. SUVmean was the most significant predictor, no other parameter significantly improved prediction (*p* = 0.0001) *Author’s Conclusions* FLT-PET parameters can predict overall survival in previously untreated patients. Patients without identifiable FLT uptake have good prognosis, while those with clearly visible pathologic FLT uptake have a significantly higher risk of death *Comments and Conclusions* The retrospective nature of the data acquisition provides class III data
Nioche et al. (2013) [76]	*Study Description* Retrospective, single center *Patient Population* 33 patients (18 HGG and 15 LGG) *Treatment Regimen* All patients underwent CT and PET imaging. In static imaging, the use of SUVmean and SUVmax derived from 18 F-FDopa PET/CT from various time ranges following acquisition in grading gliomas was assessed and compared to parameters derived from dynamic imaging. ROC curves were constructed for each parameter. A non-parametric test was used to test the significant of the difference between the areas under the ROC curves	Class III	*Results* Parameters derived from 18F-FDopa PET/CT can distinguish LG and LG tumors in recurrent gliomas. There was a significant difference in the SUVmean and max between HGG and LGG when considering all time intervals. Using a SUVmean threshold of 2.5, HG tumors could be distinguished from LG tumors with a SEN of 70%, and a SPE of 90%. While the accuracy was marginally higher in k1 derived from dynamic imaging compared to static imaging, this difference was not significant *Author’s Conclusions* 18F-FDOPA is useful in differentiating HGG and LGG. LGG and HGG can be distinguished in recurrent gliomas using static 18F-FDOPA PET. Discrimination was slightly but not significantly improved when dynamic images were acquired and analyzed *Comments and Conclusions* The retrospective nature of this study yields class III data
Bisdas et al. (2013) [64]	*Study Description* Prospective, single center *Patient Population* 28 consecutive glioma patients *Treatment Regimen* All patients underwent MR-PET and MRS. Patients were divided into 4 groups based on the relation between the Met uptake area and the increased metabolite ratio. The utility of parameters derived simultaneous MRS and MR-PET in the grading of gliomas with indeterminate conventional imaging findings was studied. The role of this technique in examining the spatial distributions of metabolic changes in glioma was also studied. 2-sided Mann–Whitney test was used to make comparisons between parameters. Spearman’s correlation was used to assess for correlations between parameters from both imaging methods	Class III	*Results* High T/N Met uptake ratio does not always spatially correlate with Cho/NAA. In 10% of the lesions, increased Met uptake area had at least 50% overlap with the area of increased Cho/Naa. 14% of the lesions had less than 50% overlap. In 21% of the lesions, the increased Met uptake region had no special relationship with the hot lesions in the MRS map. Spearman rank test showed significant correlations between MET uptake and Cr/NAA ratio (ρ = 0.59, *p* = 0.015) as well as between Cho/NAA and Cr/NAA ratios (ρ = 0.79, *p* = 0.0002). There was no accumulation of Met in 54% of the lesions *Author’s Conclusions* Metabolic mapping is feasible using simultaneous MR-PET imaging. High T/N MET uptake radio, which is indicative of proliferating tumor cell populations, does not always spatially correlate with neuronal cell loss and cell membrane proliferation (Cho/NAA) seen in MRS *Comments and Conclusions* As there is no contemporaneous or carefully matched control population this represents class III data
Galldiks et al. (2013) [73]	*Study Description* Prospective, single institution *Patient Population* 27 histologically proven LGG patients *Treatment Regimen* All patients underwent MR imaging and dynamic F-FET PET examinations. The value of FET-PET uptake as an indicator for malignant progression of LGG was evaluated. Student t test and Wilcoxon test were used to compare FET-PET parameters before and after malignant progression. FET-PET parameters were assessed using ROC curves	Class III	*Results* TBRmax and mean at baseline were significantly lower in LGG patients with progression than in those without progression (*p* = 0.04). In patients with histologically proven progression, TBRmax and mean values were significantly increased compared to baseline values(*p* < 0.001). An increase of 33% in TBRmax was determined as an optimal cutoff to identify progression to HGGs (SEN: 72%, SPE: 89%, PPV: 93%, NPV: 62%). Changes in FET-parameters were an independent factor of malignant progression (*p* = 0.013) in multiple regression analysis *Author’s Conclusions* FET-PET provides valuable diagnostic information with higher diagnostic accuracy than conventional MR imaging for the noninvasive detection of spontaneous malignant tumor progression in LGG patients. Repeated FET-PET may be helpful for treatment decisions *Comments and Conclusions* The lack of randomization or identification of a contemporaneous cohort of patients imaged without PET leaves this as class III data

*MET-PET* positron emission tomography (PET) imaging with 11C methionine, *TAC* time-activity curves, *ANOVA* analysis of variance, *SUV* standardized uptake volume, *TTP* time to peak, *SEN* sensitivity, *SPE* specificity, *FET* O-(2-18F-fluoroethyl)-l-tyrosine, *ROC* receiver operating characteristics, *LGG* low grade glioma, *RANO* response assessment in neuro oncology, *WHO* World Health Organization, *TBR* tumor to brain ratio, *PH* proportional hazards, *TCR* tumor/choroid plexus uptake ratio, *PFS* progression free survival, *OS* overall survival, *AUC* area under the curve, *FDOPA* amino-acid-analogue L-3,4-dihydroxy-6-18F-fluorophenyl-alanine, *MRSI* magnetic resonance spectroscopic imaging, *PPV* positive predictive value, *NPV* negative predictive value, *HGG* high grade glioma, *MRI* magnetic resonance imaging, *DSC* dynamic susceptibility contrast, *DWI* diffusion weighted imaging, *ADC* apparent diffusion coeffecient, *CBV* cerebral blood volume, *IDH* isocitrate dehydrogenase, *pMRI* perfusion MRI, *dMRI* difusion MRI, *cMRI* conventional MRI, *DLGG* Diffuse low-grade glioma, T/*N* tumor/normal brain ratio, T/*S* tumor/striatum ratio, *IDSI* isotropic diffusion spectrum imaging, *AMT* α[11C]methyl-L-tryptophan, *GBM* glioblastoma multiforme, *TMZ* temozolomide, *FLT* flurothymidine, *Cho* choline, *Naa* N-acetyl aspartate, *MRS* magnetic resonance spectroscopy

#### Results of individual studies, discussion of study limitations and risk of bias

Balos et al. found in a study with 14 grade II and 8 grade III gliomas, Choline/N-acetyl aspartate(Cho/NAA) ratio was able to distinguish between two groups [11]. Raschke et al. were able to achieve an accuracy of 86% using MRS for differentiating between grade II and grade IV gliomas [61]. Naveed et al. explored the utility of MRS in grading of oligodendroglial tumors along with perfusion, permeability and ADC to find no statistical difference between grade II and grade III lesion based on choline/creatine, and choline/NAA [15]. Yoon et al. demonstrated significant differences in Choline to Creatine and Lipid-Lactate to Creatine ratios between low- and high-grade gliomas with 63 and 70% accuracy, respectively. When the ratios were used together, the accuracy improved to 81.6% [35]. Other multiparametric approaches by Caulo et al., Fudaba et al. and Bisdas et al. demonstrate utility of MRS metabolite ratios in differentiating between low and high grade gliomas [27, 28, 36]. Tong et al. demonstrated the role of dynamic MRS and difference in Choline to NAA ratio to differentiate between low and high grade gliomas [62]. Bund et al. found that the nCho/Cr and nCho/NAA rations were significantly higher in high- than in low-grade gliomas in a study of 53 total subjects [63]. Previous work by Guillevin et al. provided supporting data for these studies demonstrating that proton magnetic resonance spectroscopy measurements of the choline/creatine ratio, resonance of lactates and resonance of free lipids correlates with Ki-6 7 immunohistochemistry in diffuse low grade gliomas [64].

For prediction of PFS after first recurrence, Neill et al. performed MRS imaging in 122 patients with baseline diagnosis of grade II gliomas, who presented with recurrence (41% Grade II, 43% Grade III and 15% Grade IV). The authors found that after adjusting for age, EOR and subsequent treatment, Cho/NAA and Lactate were higher in patients with poorer PFS [40]. This data supports a previously published single cohort study showing the value of proton spectroscopy parameters over tumor volume alone in predicting progression of grade II gliomas during treatment with temozolomide [65].

A single study evaluating the role of MRS for post treatment monitoring was identified. Lotumolo et al. compared MRS and ADC to evaluate for tumor progression in 32 LGGs treated only by radiation therapy with RANO criteria used a gold standard for comparison. Standard and advanced MRI was performed 3 months after completion of RT and then subsequently at every 4–6 month intervals. The authors found higher accuracy for ADC (87.5%) as compared to Cho/NAA (62.5%), NAA/Cr (43.7%) and Cho/Cr (50%) [41].

#### Synthesis

There were only a handful of class III articles on MR spectroscopy that demonstrated ability to differentiate between low- and high-grade gliomas. No MRS studies to assess IDH status were eligible to be included in this review. A single study on MRS found utility in performing MRS for post treatment monitoring. A single study identified the utility of performing diffusion, perfusion and MRS for prediction of progression free survival after first recurrence in LGGs. Given the class III evidence, a level III recommendation for role of MR spectroscopy in WHO Grade II diffuse gliomas can be made that MR spectroscopy be performed for grading of this information is not available through other techniques.

None of the studies were able to give information on delineation of margins in LGGs.

*Q2: In adult patients with suspected or histologically proven WHO Grade II diffuse glioma, does molecular imaging using amino acid PET tracers provide superior assessment of tumor grade, margins, progression, treatment-related effects, and prognosis compared to standard neuroimaging?*

### Study selection and characteristics

After full text review 18 studies met the inclusion criteria for inclusion in this guideline (Table 7).

### Results of individual studies, discussion of study limitations and risk of bias

The studies that met inclusion criteria regarding amino acid PET imaging provide class III data regarding the use of advanced preoperative imaging techniques based on their retrospective nature, or prospective nature with no meaningful contemporaneous, historical control or validation set. The studies are discussed by tracer molecule.

#### O-(2- [18F]-fluoroethyl)-L-tyrosine (18F-FET) positron emission tomography (PET): FET PET

A total of six qualifying manuscripts dealt with FET as a tracer. In two, use of this compound was helpful in preoperative studies to provide predictive information separating low grade gliomas from high grade gliomas, but in neither paper could oligodendroglioma be separated from astrocytoma. Bette et al. noted elevated FET uptake in LGG, but there was no correlation with IDH status, or survival [66]. Gempt et al. found low grade gliomas had a T/N ratio (tumor/normal) just over half that seen in HGG, 1.65 vs. 3.14, respectively, and this was deemed as likely significant (*p* < 0.001) [67].

In four of the six manuscripts, FET PET was used to assess prognosis. In the manuscript by Gempt et al. studying 152 patients with low grade gliomas prior to surgery, no deaths were recorded during the follow-up period for those with a T/N ratio < 1.6, while median survival for T/*N* > 3 was 14.0 months (*p* < 0.001) [67]. Jansen et al. also found FET PET to have prognostic value noting that in patients with FET-positive gliomas, increasing time to peak on time-activity curves presented with significantly longer progression free survival (*p* < 0.01), time to malignant transformation (*p* < 0.001), and overall survival (*p* = 0.002) [68]. Thon et al. characterized FET-PET time activity curves into three patterns which they termed homogeneous increasing, focal decreasing, and homogenous decreasing [69]. Patients with homogeneous increasing time activity curve patterns were less likely to suffer tumor progression during the time of the study follow-up and had a superior progression free survival compared to those with the homogeneous decreasing time activity curve pattern [69]. Conflicting with these three conclusions were the findings of Bette et al. where FET uptake was not correlated with survival [66]. The study to use FET PET for detection of progression produced conflicting results between different authors. Bashir et al. found that interindividual variability in FET uptake within in different WHO groups was high, and a reliable cut off value could not be determined for TBRmax and mean using ROC analysis [70]. Also trying to use FET PET to detect progression, Galldiks et al. found that in patients with histologically proven progression, TBRmax and mean values were significantly increased compared to baseline values (*p* < 0.001). An increase of 33% in TBRmax was determined as an optimal cutoff to identify progression to higher grade gliomas (sensitivity: 72%, specificity: 89%, positive predictive value: 93%, negative predictive value: 62%) [71]. None of the qualifying FET PET studies provided findings suggesting this tracer contributes significantly to identification of tumor margins assessing treatment response.

#### [18F]-fluoro-3’-deoxy-3’-L: -fluorothymidine (FLT) positron emission tomography: (FLT) PET

One study looking at FLT PET qualified for inclusion in this guideline. Belohlavek et al. scanned 41 patients with low grade gliomas prior to radiation therapy [72]. FLT-PET parameters predict overall survival in previously untreated patients with SUVmean being the most significant predictor (*p* = 0.0001). Patients without identifiable FLT uptake had good prognosis, while those with clearly visible FLT uptake in the region of tumor had a significantly higher risk of death during the observation period of this study [73].

#### L-methyl-[11]C-methionine-positron emission tomography: (MET) PET

Four studies assessing MET PET met inclusion criteria for this guideline update. In a study of MET PET by Nomura et al., there was no difference in MET-SUV between anaplastic astrocytoma, diffuse astrocytoma and normal frontal cortex. They did observe that MET SUV was higher in the initial phase and had tendency to increase more in the early phases of the time activity curve in tumors with an oligodendroglial component [74]. In contradiction to these Tietze et al. found that MET PET added little to the ability to diagnose or grade low grade gliomas beyond what could be derived from various MRI sequences [72].

Bisdas et al. found that the addition of MET PET to nCr (Creatine in tumor voxel/Creatine in the contralateral healthy voxel)values from MR spectroscopic data assisted in separating low grade gliomas from high grade gliomas, where low grade lesions were associated with lower values of MET T/N (T/*N* = tumor to normal brain uptake ratio) and nCr [74]. Santoni et al. reported that 11C-MET PET was able to provide early detection of progression of low grade to anaplastic astrocytoma with a sensitivity of 91.56% and a specificity of 95.18% [75]. None of the studies looking at MET PET assessed for this modality was as a method of delineating tumor margins or response to treatment.

#### 4-dihydroxy-6- [18F]-fluoro-phenylalanine positron emission tomography: FDOPA PET

Upon review of the literature within the search period, five manuscripts dealing with FDOPA met inclusion/exclusion criteria for use in this guideline. Three papers dealt with the use of FDOPA for determining tumor grade prior to surgery. In a study by Bund et al., it was found that, using SUVmax, oligodendroglioma could be differentiated from grade I tumors and that low grade gliomas could be differentiated from high grade gliomas [63]. In a retrospective analysis of 33 patients with gliomas, Nioche et al. studied FDOPA PET/CT. There was a significant difference in the SUVmean and max between HGG and LGG when considering all time intervals of the time activity curve. Interestingly, it appears that progressive low grade tumors that are remain LGG and can be distinguished from those that are progressing to HGG using static 18F-FDOPA PET [76]. Janvier et al. studied 31 patients including 21 low grade gliomas with F-FDOPA PET. They found that SUV-derived indices are routinely available information that enables accurate discrimination of low-grade and high-grade gliomas. The best-correlated indices were SUVmean/T/N and SUVmean/T/S with thresholds of 1.33 and 1 [77]. None of these studies were able to show the ability to separate astrocytomas from oligodendrogliomas.

Two qualifying publications dealt with FDOPA as a tool for detection low grade glioma progression during therapy. Villani et al. looked at 50 low grade gliomas with serial FDOPA PET scans to assess the ability of this modality to predict eventual tumor progression. The best predictive cut-off value for maximum standardized uptake value in predicting disease progression was 1.75, with a sensitivity, specificity, positive predictive value, and negative predictive values of 0.769, 0.824, 0.625, and 0.914, respectively [78]. Rossi-Espagnet et al. looked at a small group of 12 low grade gliomas with FDOPA PET and a series of MRI sequences. There was a significant correlation between follow-up status (stable vs progressed) and T/Nmax (*p* < 0.05) derived from FDOPA PET. At a cut-off value of 1.7, this had a sensitivity of 83% and specificity of 100% of detecting progression. Neither DWI, PWI or other MRI sequences were able to accomplish this task [42].

None of the qualifying publications looked at the use of FDOPA for definition of tumor margins, treatment response or prognosis.

#### Alpha-[11C]-methyl-L-tryptophan positron emission tomography: AMT PET

Jeong et al. studied a small group of patients (5 WHO grade II and 5 WHO grade IV gliomas) with AMT PET and MRI prior to surgery. The primary parameter measured with the PET was the isotropic diffusion spectrum imaging (IDSI) with independent component analysis (ICA) in assessing the cellularity and grade of tumor. The MRI utilized primarily FLAIR, DWI and ADC imaging for the same purposes. IDSI-derived cellularity showed a slightly higher probability to differentiate HGG from LGG compared with the DWI-ADC (SEN: 80%, SPE: 80%) [37].

#### [11C]-acetate positron emission tomography

Kim et al. carried out a reasonably sized study [11C]-acetate using PET/CT prior to surgery in 73 patients with surgically confirmed cerebral gliomas (19 grade II, 21 grade III, and 33 grade IV). Using their primary parameter of tumor/choroid plexus uptake ratio (TCR) they found significant differences in TCR were seen between the three WHO grade groups (*p* < 0.001). TCR was prognostic for PFS (*p* = 0.016) and for OS (*p* = 0.024). Thus, this technique seems to be able to assist in making the diagnosis of low grade or high grade glioma and determining prognosis in low grade gliomas [78].

### Synthesis

Recent studies on amino acid PET techniques have generated enthusiasm on their potential role in glioma imaging. In diffuse grade II gliomas, a small number of papers demonstrate the ability of these tracers to separate low grade and high grade gliomas with mixed results. These include two class III papers with FET-PET and three class III using FDOPA PET. Single manuscripts on AMT PET and[11C]-acetate PET, respectively were able to provide significant differentiation between high and WHO grade II diffuse gliomas. The ability to grade gliomas with MET-PET was suggested in two class III papers but contradicted in three other class III papers. Neither FET PET, FDOPA, AMT PET nor [11C]-acetate PET could reliably separate astrocytomas from oligodendrogliomas. In light of this evidence, a level III recommendation is made that if not already evident by MRI studies and if it would be of value to the clinician to know if a brain lesion is a low-grade glioma or high-grade glioma before surgery, then the addition of amino acid PET with FET or FDOPA as a tracer may be considered. The data on AMT and [11C]-acetate is limited to single papers and does not warrant making a recommendation. The use of MET-PET for this purpose is not currently recommended.

Use of FET PET and MET-PET for assessing low grade glioma progression during treatment resulted in conflicting class III results between publications. The ability of a standardized FDOPA PET uptake cut off values to detect progression of low-grade gliomas to a higher grade lesion was confirmed in two class III papers. In light of these data, a level III recommendation is made that if a clinician believes they need additional information beyond MRI for detection of tumor progression FDOPA PET is suggested as a useful adjunct.

Though three manuscripts provided class III data to suggest that FET PET could be of assistance in predicting low grade glioma survival and prognosis, one class III manuscript contradicted that ability. The single class III studies qualifying on FLT PET and [11C]-acetate PET found that scans with these tracers prior to surgery to be of value in assessing prognosis. Given limited data, a level III recommendation is made which states that if standard clinical prognostic parameters are unclear, and novel PET tracers are available, the clinician might consider FET PET to assist in determination of the prognosis in an individual with a WHO Grade II diffuse glioma.

The search of the amino acid PET tracers qualifying for inclusion in this guideline did not provide evidence that they can assist in definition of tumor margins. Though the qualifying literature was searched for the use of amino acid PET to assess treatment response, none of the six tracers identified were found to have addressed that endeavor.

## Discussion

Since the previous guidelines were published, a significant amount of new literature on role of advanced MRI techniques and amino acid PET imaging in assessment of low-grade gliomas has been published. The previous guidelines focused on diagnosis, biological behavior/ prognosis and imaging follow-up, while the current guidelines focus on assessing the role of specific imaging techniques in evaluation of WHO Grade II diffuse gliomas. In addition to advances in imaging, the last 10 years have also seen evolution in WHO Grade II diffuse glioma management and follow-up strategies, largely driven by the WHO classification of primary brain tumors in 2016, and more recently, in 2021. The current management strategies for low grade gliomas include surgical resection followed by radiatio*n* ± chemotherapy depending on presence of additional risk factors. Standard clinical MR imaging is essential for diagnosis, surgical planning, and immediate post-surgical assessment as well as for follow-up. This guideline update provides an overview of how advanced MRI and amino acid PET imaging can provide additional information during the entire course of therapy in a manner that can play a role in clinical decision making.

Of all the advanced MRI techniques, the most robust evidence available was for diffusion imaging. Diffusion parameters such as ADC and others are indicators of tumor hypercellularity and unsurprisingly have shown significant promise in glioma grading to differentiate low from high grade neoplasms. Furthermore, a small but significant body of literature shows that diffusion imaging has value in prediction of IDH mutation status, post treatment monitoring and recurrence prediction. The perfusion data on grading of gliomas is also robust and can be easily explained by the ability of various perfusion parameters to identify tissue changes related to tumor angiogenesis and hypervascularity. Indeed, the simplest evidence supporting this phenomenon is more frequent presence of gadolinium-based contrast enhancement in higher grade gliomas as compared to LGGs. Amino acid PET agents have physiological basis for characterization of gliomas as these agents can detect the burden of DNA replication and cellular proliferation. In theory, lower uptake of amino acid PET agents in grade II gliomas as compared to grade III and grade IV neoplasms should be a universal finding, however, inconsistencies in tracer uptake, and differences based on molecular status can make the interpretation challenging.

## Conclusions and future research

Based on this current guideline update, advanced MRI imaging techniques and amino-acid PET agents can play valuable supportive role in management of WHO Grade II diffuse gliomas. For future studies, effort needs to be directed towards critical areas where advanced imaging can play a more dominant role. Some of these areas of clinical interest are discussed here.

Grade II gliomas with IDH-WT status are associated with unfavorable outcomes as compared to IDH-mutant neoplasms. While IDH status is confirmed on tissue analysis after surgery, having a-priori knowledge before surgery will help surgeon pursue more aggressive resection strategies in suspected IDH-WT lesions. The diffusion and perfusion data on IDH status determination is limited, without clear evidence to support their implementation in clinical practice. While there is significant body of literature that demonstrates utility of 2hydroxyglutarate MRS for IDH detection in gliomas, these papers consider all glioma grades together and did not qualify for use in this guideline [79].

Nearly all LGGs eventually recur at some point during the post treatment phase which can be as short as 2 years or as long as 2 decades. The first sign of tumor progression is new area of enhancement or new signal abnormality on the MRI scan. About 50% of LGGs recur as grade II, 30% as grade III and 20% recur as grade IV gliomas. Given the unpredictable timeline of recurrence, there is a need for an imaging tool that can pre-date the standard MRI changes to predict recurrence so that early changes in follow-up regimen and treatment plan can be made. Again, the evidence from diffusion imaging studies is the most promising in this regard as compared to other advanced MRI techniques. More studies with perfusion and MRS are needed to further assess the potential of these techniques in post-treatment monitoring and recurrence prediction. Similarly, despite significant strengths, amino acid agents need more assessment in WHO grade II diffuse gliomas to assess their role in treatment response assessment.

## Data Availability

No datasets were generated or analysed during the current study.

## Declarations

### Disclaimer of liability

This clinical systematic review and evidence-based guideline was developed by a physician volunteer task force as an educational tool that reflects the current state of knowledge at the time of completion. Each chapter is designed to provide an accurate review of the subject matter covered. This guideline is disseminated with the understanding that the recommendations by the authors and consultants who have collaborated in their development are not meant to replace the individualized care and treatment advice from a patient’s physician(s). If medical advice or assistance is required, the services of a competent physician should be sought. The proposals contained in these guidelines may not be suitable for use in all circumstances. The choice to implement any particular recommendation contained in these guidelines must be made by a managing physician in light of the situation in each particular patient and on the basis of existing resources.
